# Drug-transporter mediated interactions between anthelminthic and antiretroviral drugs across the Caco-2 cell monolayers

**DOI:** 10.1186/s40360-017-0129-6

**Published:** 2017-05-04

**Authors:** Gabriel Kigen, Geoffrey Edwards

**Affiliations:** 1 Department of Pharmacology and Toxicology, Moi University School of Medicine, P.O. Box 4606, 30100 Eldoret, Kenya; 2 Department of Molecular and Clinical Pharmacology, University of Liverpool, Liverpool, L69 3GE UK

**Keywords:** Antiretroviral, Antiparasitic, Drug interactions, Caco-2 cell monolayers, Drug transport, Intestinal epithelium, TEER

## Abstract

**Background:**

Drug interactions between antiretroviral drugs (ARVs) and anthelminthic drugs, ivermectin (IVM) and praziquantel (PZQ) were assessed by investigating their permeation through the Caco-2 cell monolayers in a transwell. The impact of anthelminthics on the transport of ARVs was determined by assessing the apical to basolateral (AP → BL) [passive] and basolateral to apical (BL → AP) [efflux] directions alone, and in presence of an anthelminthic. The reverse was conducted for the assessment of the influence of ARVs on anthelminthics.

**Methods:**

Samples from the AP and BL compartments were taken at 60, 120, 180 and 240 min and quantified either by HPLC or radiolabeled assay using a liquid scintillating counter for the respective drugs. Transepithelial resistance (TEER) was used to assess the integrity of the monolayers. The amount of compound transported per second (apparent permeability, *P*app) was calculated for both AP to BL (*P*app_AtoB_), and BL to AP (*P*app_BtoA_) movements. Samples collected after 60 min were used to determine the efflux ratio (ER), quotient of secretory permeability and absorptive permeability (*P*appBL-AP/PappAP-BL). The reverse, (*P*appAP-BL/PappBL-AP) constituted the uptake ratio. The impact of SQV, EFV and NVP on the transport of both IVM and PZQ were investigated. The effect of LPV on the transport of IVM was also determined. The influence of IVM on the transport of SQV, NVP, LPV and EFV; as well as the effect PZQ on the transport of SQV of was also investigated, and a two-tailed *p* value of <0.05 was considered significant.

**Results:**

IVM significantly inhibited the efflux transport (BL → AP movement) of LPV (ER; 6.7 vs. 0.8, *p* = 0.0038) and SQV (ER; 3.1 vs. 1.2 *p* = 0.00328); and increased the efflux transport of EFV (ER; 0.7 vs. 0.9, *p* = 0.031) suggesting the possibility of drug transporter mediated interactions between the two drugs. NVP increased the efflux transport of IVM (ER; 0.8 vs. 1.8, *p* = 0.0094).

**Conclusions:**

The study provides in vitro evidence of potential interactions between IVM, an anthelminthic drug with antiretroviral drugs; LPV, SQV, NVP and EFV. Further investigations should be conducted to investigate the possibility of in vivo interactions.

**Electronic supplementary material:**

The online version of this article (doi:10.1186/s40360-017-0129-6) contains supplementary material, which is available to authorized users.

## Background

The sub-Saharan Africa still leads in the prevalence of Human immunodeficiency virus infection and acquired immune deficiency syndrome (HIV/AIDs), malaria, tuberculosis and helminthic infections. HIV positive patients are thus likely to be co-infected with any of the diseases, and the co-administration of anthelminthics and ARVs is not uncommon [1]. This can give rise to drug-drug interactions (DDIs) which are likely to alter the therapeutic outcome of each of the drugs. These interactions may result in an increase or decrease in the plasma concentrations of the drugs thereby increasing the risk of toxicity or development of resistance amongst other adverse effects. This may in some instances require dosage adjustments [2–4]. The knowledge of any potential interactions between these drugs is therefore important in optimization of HIV therapy [5, 6]. The potential mechanisms of the drug interactions include modulation by drug transporters (both efflux and influx), and inhibition or induction of drug metabolizing enzymes [6–14].

Several drugs are substrates and/or inhibitors of these efflux transporters and metabolic enzymes, especially CYP 3A4 [15, 16]. Among the ARVs, protease inhibitors (PIs) are known to be substrates of P-gp, ABCC 1 and ABCC 2 [3, 12, 17, 18]. Saquinavir (SQV) and lopinavir (LPV) are substrates and inhibitors of drug transporters [19, 20]. Nucleoside reverse transcriptase inhibitors (NRTIs) and non-nucleoside reverse transcriptase inhibitors (NNRTIs) have also been characterized as substrates for drug transporters [21–24]. Newer NNRTIs such as etravirine have been reported to induce ABC transporters especially BCRP/ABCG2, and therefore potential for drug interactions with co-administered drugs that are substrates for these transporters [25]. Rilpivirine has also been reported to induce and inhibit several relevant drug-metabolizing enzymes and drug transporters albeit with less potential for drug interactions owing to low plasma concentrations [26].

PZQ and IVM are some of the most widely used anthelminthic drugs. PZQ is mainly used to treat schistosomiasis, whereas IVM is used in the treatment of lymphatic filariasis [27, 28]. Both diseases are endemic in developing countries with schistosomiasis afflicting over 200 million people [29, 30], and lymphatic filariasis having a global prevalence of over 120 million people with an estimated 1.3 billion at risk [31, 32]. IVM has been characterized as a substrate and inhibitor of P-gp [33–35]. IVM interacts with P-gp modulators [36, 37], and the inhibition of P-gp has been described as a potential strategy to counter the emerging resistance to IVM [38]. PZQ has not been conclusively characterized with regards to drug transporter specificity [39, 40], but from the available data, it is known to be metabolized by cytochrome P450 isoenzymes (CYP), mainly CYP2B1, CYP3A4, CYP2C9 and CYP2C19. It therefore has potential to interact with drugs which are inhibitors or inducers of these enzymes [41, 42]. Enzyme inducers such as carbamazepine, phenytoin and rifampicin reduce PZQ plasma levels, while ketoconazole, an enzyme inhibitor significantly increases its concentration [41, 43]. Previous researchers on transport of antiparasitic drugs along the Caco-2 cell monolayers (CCM) have also reported that PZQ is an inhibitor of P-gp without being a substrate [40]. However, it is not clear how the investigators concluded that this was specifically mediated by P-gp using the CCM model since CCM expresses several other transporters [44]. From the research conducted earlier in our laboratories we have demonstrated that PZQ is neither a substrate nor an inhibitor of P-gp in CEM T-lymphoblastoid cells [45].

**Fig. 1:**
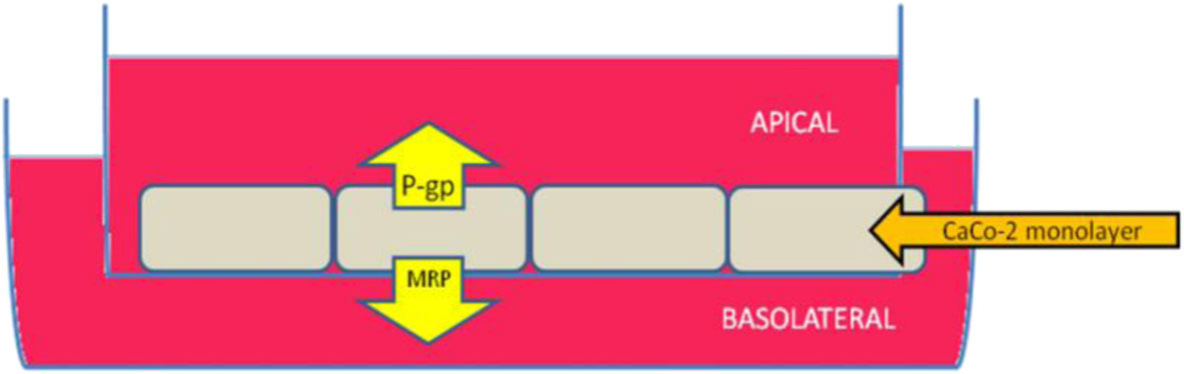
An illustration of a transwell depicting the growing Caco-2 cells including the compartments and the positions of the transporters

The majority of drugs in use are orally administered, and their absorption from the gastrointestinal tract is pivotal for their therapeutic success [46]. The ability of a drug to cross the intestinal wall in order to reach portal circulation is to a large extent dependent on its permeability coefficient [47]. The Caco-2 cell model provides a simple and reliable method to assay in vitro permeability of drugs [48–50]. The permeability of drugs through the CCM correlates well with in vivo absorption in humans thus making the CCM an invaluable analytical tool in the screening of orally administered drugs [51–54]. CCM are derived from human colonic adenocarcinoma and have morphological as well as functional similarities to intestinal (absorptive) enterocytes [44, 55–57]. They have adherent properties and therefore form a monolayer with tight junctions which prevent paracellular diffusion so that drugs or other solutes can only pass through the cell, as illustrated in the cartoon (Fig. 1). This in addition results in development of cell polarity, and the efflux transporter P-glycoprotein (P-gp) has been shown to be localized on the apical brush border, approximately 20 microns above the base of the cells [58], while certain efflux transporters such as multidrug resistance-associated proteins (MRPs) are expressed on the basolateral side of the monolayer [59, 60]. Apart from P-gp and MRPs, CCM express a wide array of transporters (efflux and influx) as well as metabolic enzymes, thus making them suitable for the study of drug-drug interactions based on the permeability of drugs through the monolayers [20, 44, 55, 56, 61, 62].

The permeation of drugs through the monolayers allows the study of the major absorptive mechanisms for drugs, such as passive transcellular transport and carrier-mediated influx as well as efflux mechanisms [63]. In this system, the passage of drugs from apical (AP) to basolateral (BL) compartment is attributable to passive diffusion and occurs at a lower rate, whereas the BL to AP passage occurs by active transport, presumed to be mediated by transporters [20, 44, 64]. The carrier-mediated transport is a saturable process, which raises a possibility that when two drugs are co-administered they may compete for a transporter (influx or efflux) which would lead to drug interactions leading to lower or higher exposure than when dosed alone [20, 65]. The cells are grown on a porous membrane and form differentiated monolayers after about 20 days. The membranes are polarized and evaluation of the monolayers can be performed by measuring the transepithelial resistance (TEER), using a volt-ohm meter equipped with electrodes placed in the upper and lower chambers of the insert. TEER increases with culture reaching a maximum in about 10-15 days [66–68], and depends on the number of cells seeded, plus the surface area of the filter. TEER values range from 150 to 1600 ohm.cm^2^ as compared to human ileum which is about 50 ohm [69].

To date there is scant information from the literature regarding the interactions between ARVs and anthelminthics despite the likelihood of their co-administration in tropical regions owing to their geographic overlaps. The aim of the study was therefore to assess the potential DDIs between ARVs and the anthelminthic drugs; PZQ and IVM. In the study, CCM was used to evaluate potential interactions between ARVs and anthelminthics. The impact of the anthelminthics on the ARVs transport was determined by assessing the AP → BL and BL → AP directions alone, and in presence of an anthelminthic, and the reverse was conducted for the assessment of the influence of ARVs on anthelminthics. Quantification was performed using an HPLC method described earlier [45], or radiolabeled assay using a liquid scintillating counter. PZQ and IVM were used as prototypes for the anthelminthics, and PIs, saquinavir (SQV) and lopinavir (LPV) as well as NNRTIs, efavirenz (EFV) and nevirapine (NVP) as prototypes of ARVs. The impact of SQV, EFV and NVP on the transport of PZQ as well as the impact of PZQ on the transport of SQV was determined. The influence of SQV, NVP, LPV and EFV on the transport of IVM, and IVM on the transport of SQV, NVP, LPV and EFV were also investigated.

## Methods

### Equipment

The HPLC consisted of a Dionex (Dionex Softron GmbH, Germany) HPLC system with a P 680 pump, an ASI-100 automated sample injector and a UVD 1704 detector. A 250μl injector with a 20μl loop was used. Reversed-phase-liquid chromatography was carried out using a Hypurity™ C_18_ analytical column, 5μm x 4.6mm (Thermo Electron Corporation, Runcorn, UK 22105-154630). A column guard (Thermo electron 60140-412) was used to protect the analytical column. The ultraviolet detector was set to monitor the 215 nm wavelength. A Packard Tri-Carb Liquid Scintillation Analyzer model 1900 TR (Packard instrument Co.) was used for radioactivity counting. A Millicell Electrical Resistance System (Fisher Scientific, Leicestershire, UK) was used for measuring the transepithelial electric resistance (TEER). Transwells (six-well transwell polycarbonate tissue culture treated plates, 4.67 cm^2^, 24 mm diameter; 0.4 μm pore size) were purchased from Corning life Sciences (Costar High Wycombe, Bucks; UK).

### Materials

The human colon adenocarcinoma cell line, Caco-2 was purchased from European collection of cell cultures (ECACC No. 286010202), and the cells were counted using a Nucleo Counter (ChemoMetec, Denmark) cell counter. SQV was provided by Roche Discovery (Welwyn Garden City, UK), LPV by Abbott Laboratories (Chicago, USA), EFV by Dupont Bristol Myers Squibb (New Brunswick, NJ, USA), and NVP by Boehringer Ingelheim (Berkshire, UK). Radiolabelled SQV (^3H^ SQV), LPV (^3^H LPV), EFV (^14^C EFV) and NVP (^3^H NVP) were purchased from Moravek Biochemicals (Brea California, USA). ^3^H IVM was kindly donated by Dr Iain Gardner of Pfizer (Sandwich, Kent, UK). PZQ, CLZ, DMEM, HBSS, FBS, DMSO and Trypsin-EDTA solution were purchased from Sigma Aldrich (Poole, UK). ACN and MeOH were purchased from VWR Laboratory Supplies (Poole, UK) whereas diethyl ether was purchased from Fisher Scientific, (Loughborough, UK). Ultima Gold liquid scintillation cocktail was obtained from Packard (Groningen, Netherlands). All the other chemicals used were of analytical or HPLC grade. Deionised water used to prepare the solutions or mobile phase, and was purified in an Elga DV 25 pure lab option system (Elga, High Wycombe, Bucks, and UK).

### Caco-2 cell lines

#### Cell culture

Caco-2 cells were cultured in Dulbecco's Modified Eagle Medium (DMEM) supplemented with fetal bovine serum (FBS) [15%v/v]. The cells were grown and routinely seeded in tissue cultured treated 162 cm^2^ flasks in a humidified chamber (37°C, 10% CO_2_ incubator) and harvested by regular trypsinization. The medium was changed every 2 to 3 days until the confluence of the cell monolayer was achieved. Trypsinization involved decanting the media, followed by washing twice with 6 ml of Hanks' Balanced Salt Solution (HBSS), and the detachment of the monolayer by addition of 4 ml of trypsin EDTA. The cells were then incubated for 10 min. The resulting suspension was then centrifuged (2000 *g* × 5 min, 4°C) and supernatant removed. The resulting pellet was then re-suspended in 20 ml of fresh DMEM (+15% FBS), 10 ml transferred two new flasks; and each made to 20 ml.

### Storage of Caco-2 cells

The cells were trypsinised as described earlier after attaining confluence. The pellets were then re-suspended in DMEM (15%v/v), counted using a nucleocounter and centrifuged (2000 *g* × 5 min, 4°C). The cells were then re-suspended in warm FBS (FBS + 10% DMSO), mixed thoroughly and made up to a concentration of 5 × 10^6^ cells per ml. 1 ml of the cell suspension was then transferred to pre-labelled 1.5 ml cryovials and frozen at -80°C for use as and when required. The viable semi-frozen cells were thawed by placing the cryovials rapidly in a waterbath (37°C), or by simply holding in the vials in the hands for a few minutes and re-suspending the pellets in 9 ml DMEM (+15%FBS) followed by culturing.

### Determination of drug transport in Caco-2 cells

#### Cell seeding

The monolayers used were of between passage 20 and 65 after 15 days of growth. Each experiment was performed in duplicate using two six well-transwell culture plates. The cells were trypsinized as described earlier, and after centrifugation the pellet re-suspended in fresh DMEM (+15%), and the cells counted using a cell counter. A volume of DMEM was added enough to give a cell count of 2 × 10^6^ cells per ml, and cells were seeded on the transwell culture plates at a density of 2 × 10^4^ cells/cm^2^ (~ 100,000 cells per well, since insert membrane growth area = 4.67 cm^2^). The plates were then incubated at 37°C and 10% CO_2_ in a humidified chamber and the media changed every 2-3 days, by aspirating using a suction pump and replacing with an equal volume of DMEM. Transport experiments were conducted 15 to 20 days after seeding. The TEER across the cell monolayers was monitored using a Millicell-ERS in order to assess cell monolayer integrity and the monolayers considered appropriate for the experiment when the TEER values were typically above 500 cm^2^ [49, 70].

#### Transport experiments

Prior to transport studies, each monolayer was washed and equilibrated with the transport medium (DMEM without FBS). The medium was removed from all AP and BL compartments of the transwells and replaced with 2 ml of the transport medium (DMEM alone), to both compartments and equilibrated for 1 h (37°C, 10% CO_2_ incubator), after which the TEER was re-assessed and labeled. The medium was then removed from both compartments and replaced with an equal volume of pre-warmed medium containing the compound of interest at the appropriate concentration. For the AP → BL transport, 2 ml of medium containing the desired drug was placed in the AP chamber and 2 ml of the medium alone in the BL chamber, whereas 2 ml of medium containing the drug was placed in the BL and 2 ml of medium on the AP chamber for the BL → AP transport [71]. The effect of the second drug was then assessed by adding the medium containing the original drug and the drug under study to the AP side for AP → BL transport with the medium containing the original drug alone in the BL chamber, and vice-versa for the BL → AP transport. Transport in each direction was done in triplicate. The transwell plates were then incubated (37°C, 10% CO_2_ incubator), and 100μl of the samples from the AP and BL compartments were taken at 60, 120, 180 and 240 min and quantified either by HPLC method described earlier [45], or by the use of a liquid scintillating counter depending on the drug under study. The HPLC method involved liquid - liquid extraction followed by ultra-high performance liquid chromatography using a Hypurity C_18_ column and ultraviolet detection set at a wavelength of 215 nm. The mobile phase consisted of ammonium formate, acetonitrile and methanol (57:38:5 v/v). Separation was facilitated via isocratic elution at a flow rate of 1.5 ml/min. For HPLC assays, the concentration used for each drug was 20μg/ml. The concentrations that offered the best detection were selected for radiolabeled assay. Table 1 summarizes the type of assay and the various concentrations used in each experiment. HPLC was used in the assay of interactions between SQV/PZQ, IVM/SQV, EFV/PZQ and NVP/PZQ; while radiolabeled assay was utilized in the investigations of the interactions between PZQ/SQV, SQV/IVM, NVP/IVM, IVM/NVP, LPV/IVM, IVM/LPV, EFV/IVM and IVM/EFV. The integrity of the CCM during the experiment was monitored by measuring the TEER at the beginning (0 min) and the end of the experiment (240min).

**Table 1:** Concentration and type of assay used for each experiment

			Concentrations used			Quantification method
	A	B	Drug	μg/ml	μM	
1	PZQ	SQV	PZQ	20	64	HPLC
			SQV	20	29.8	
2	SQV	PZQ	[^3^H] SQV		1.07	Radiolabeled assay
			PZQ	20	64	
3	IVM	SQV	[^3^H] IVM		0.01	Radiolabeled assay
			SQV	20	29.8	
4	SQV	IVM	SQV	20	29.8	HPLC
			IVM	20	22.75	
5	PZQ	EFV	PZQ	20	64	HPLC
			EFV	20	63.4	
6	PZQ	NVP	PZQ	20	64	HPLC
			NVP	20	75.1	
7	IVM	NVP	[^3^H] IVM		0.01	Radiolabeled assay
			NVP	20	75.1	
8	NVP	IVM	[^3^H] NVP		1.5	Radiolabeled assay
			IVM	20	22.75	
9	IVM	LPV	[^3^H] IVM		0.02	Radiolabeled assay
			LPV	20	31.81	
10	LPV	IVM	[^3^H] LPV		1.5	Radiolabeled assay
			IVM	20	22.75	
11	IVM	EFV	[^3^H] IVM		0.01	Radiolabeled assay
			EFV	20	63.34	
12	EFV	IVM	[^14^C] EFV		0.01	Radiolabeled assay
			IVM	20	22.75	

Drug A Drug whose transport is under investigation

Drug B Interacting drug

#### Apparent permeability

The results were expressed as apparent permeability coefficient (*P*app, unit: cms^-1^), the amount of compound transported per second. *P*app values were calculated for both AP to BL (*P*app_AtoB_), and BL to AP (*P*app_BtoA_) movement of the compound. *P*app was calculated using the following equation [52, 72]:

- *P*app (cm/s) = (dQ/d*t*) x (1/(AC_O_)
- dQ/dt = Steady-state flux (dpm s^-1^ or μmol s^-1^)
- A = Surface area of the filter (cm^2^)
- C_O_ = Initial concentration in the donor chamber (dpm litre ^-1^ or μM)

The quotient of secretory permeability and absorptive permeability (*P*appBL-AP/PappAP-BL) constitutes the efflux ratio, while the reverse (*P*appAP-BL/PappBL-AP) is the uptake ratio [71]. This calculation requires that the receiver concentration should not exceed 10% of the donor concentration, and therefore was only applied for the samples taken at 60 min. The permeability is a saturable process and depends on several physiological conditions such as accumulation, pH, and lipophilicity (sink conditions), which have an effect on *P*app values with incubations over longer periods of time [71, 73]. In order to assess the potential interactions, ER of a respective drug alone was compared to that in presence of a second drug under investigation. Samples of both drugs collected after 60 min were used to investigate the trends over a period of 4 h.

### Statistical analysis

The results were presented as mean ± standard deviation (SD) of three experiments with 95% confidence intervals for differences between the means where appropriate. The analysis of the transport results obtained after 60 min was performed using a two-way analysis of variance (ANOVA). A two-tailed *p* value of <0.05 was accepted as being significant.

## Results

**Fig. 2:**
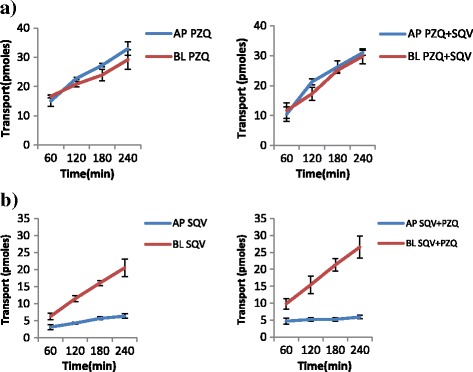
Influence of SQV on the transport of PZQ (**a**), and PZQ on the transport of SQV (**b**) across the Caco2 cell monolayers over a 4h period. The results are expressed as mean ± S.D (*n* = 3). **a** Cumulative transport of PZQ alone, and in presence of SQV. **b** Cumulative transport of SQV alone and in presence of PZQ

**Fig. 3:**
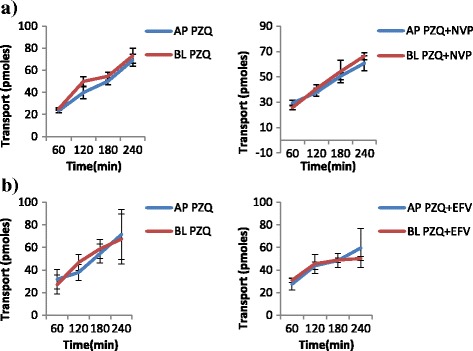
Influence of NVP (**a**) and EFV (**b**) on the transport of PZQ. **a** Cumulative transport of PZQ alone, and in presence of NVP. **b** Cumulative transport of PZQ alone, and in presence of EFV

**Fig. 4:**
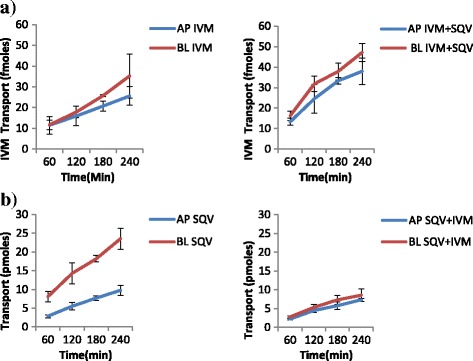
Influence of SQV on the transport of IVM **a**, and IVM on SQV transport **b. a** Cumulative transport of [^3^H] IVM alone, and in presence of SQV. **b** Cumulative transport of SQV alone, and in presence of IVM

**Fig. 5:**
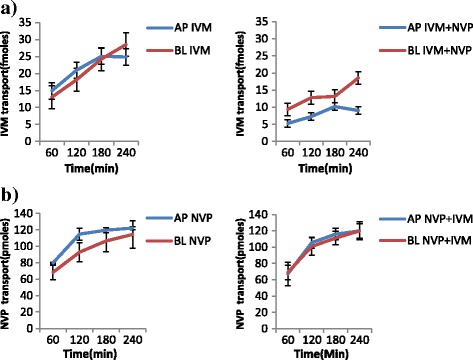
Influence of NVP on the transport of IVM **a**, and IVM on NVP transport **b. a** Cumulative transport of [3H] IVM alone, and in presence of NVP. **b** Cumulative transport of [^3^H] IVM alone, and in presence of NVP

**Fig. 6:**
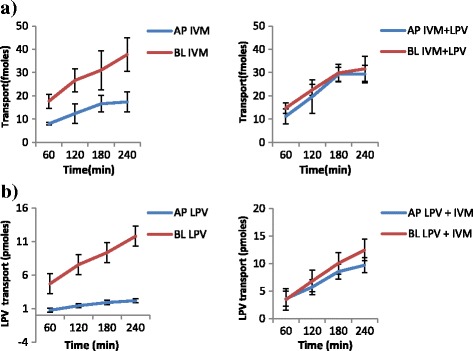
Influence of LVP on the transport of IVM **a**, and IVM on LVP transport **b. a** Cumulative transport of [^3^H] IVM alone, and in presence of LPV. **b** Cumulative transport of [^3^H] LPV alone, and in presence of IVM

**Fig. 7:**
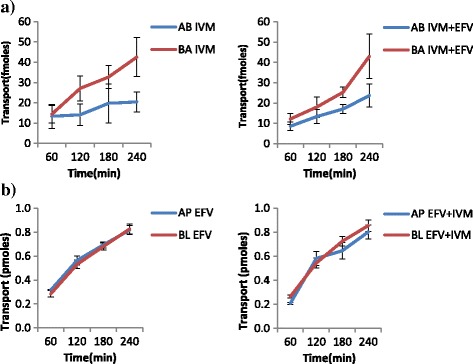
Influence of EFV on the transport of IVM **a**, and IVM on EFV transport **b**. **a** Cumulative transport of [^3^H] IVM alone, and in presence of EFV. **b** Cumulative transport of [^14^C] EFV alone, and in presence IVM

The results of the transport experiments are summarized in Table 2. IVM significantly inhibited the efflux transport (BL → AP movement) of LPV (ER; 6.7 vs. 0.8, *p* = 0.0038) and SQV (ER; 3.1 vs. 1.2 *p* = 0.0328). It also increased the efflux transport of EFV (ER; 0.7 vs. 0.9, *p* = 0.031) suggesting the possibility of drug transporter mediated interactions between the two drugs. NVP increased the efflux transport of IVM (ER; 0.8 vs. 1.8, *p* = 0.0094). Figs. 2, 3, 4, 5, 6 and 7 illustrate the trends of interactions between the respective drugs over a period of 4 h. Additional files 1, 2, 3, 4, 5, 6, 7, 8, 9, 10 show the details of how the ER values of each experiment were computed from the obtained results.

**Table 2:** Summary of the cumulative transport results showing the mean ER values after 60 min

			*P*app (10^6^ cm/s)		Mean ER value	SD	*p* value	Comments
			AP → BL	BL → AP				
1	A	PZQ	7.6	8.6	1.14	0.17	0.5008	No significant influence on PZQ transport
	B	PZQ + SQV	5.0	6.6	1.34	0.27		
	A	SQV	6.6	10.8	3.55	1.72	0.6796	No significant influence on SQV transport
	B	SQV + PZQ	18.3	27.0	2.65	1.57		
2	A	PZQ	28.1	28.6	1.03	0.19	0.0964	No significant influence on PZQ transport
	B	PZQ + NVP	37.2	30.5	0.82	0.08		
	A	PZQ	35.0	30.3	0.87	0.17	0.4676	No significant influence on PZQ transport
	B	PZQ + EFV	35.1	39.2	1.19	0.18		
3	A	IVM	7.4	5.9	0.86	0.18	0.2007	No significant influence on IVM transport
	B	IVM + SQV	6.4	6.3	0.99	0.16		
	A	SQV	6.9	21.1	3.05	0.46	0.0328	Potential for interaction; efflux transport of SQV inhibited
	B	SQV + IVM	6.2	7.7	1.24	0.14		
4	A	IVM	14.1	11.3	0.80	0.07	0.0094	Potential for interaction; efflux transport of IVM increased
	B	IVM + NVP	4.8	8.6	1.76	0.13		
	A	NVP	42.7	39.5	0.91	0.12	0.6891	No significant influence on IVM transport
	B	NVP + IVM	39.4	36.6	0.98	0.17		
5	A	IVM	4.5	13.2	2.89	0.50	0.1822	No significant influence on IVM transport
	B	IVM + LPV	6.7	9.5	1.58	0.62		
	A	LPV	4.8	32.1	6.72	0.54	0.0038	Potential for interaction; efflux transport of LPV inhibited
	B	LPV + IVM	15.9	12.3	0.78	0.10		
6	A	IVM	7.6	8.9	1.19	0.15	0.4280	No significant influence on IVM transport
	B	IVM + EFV	4.7	7.3	1.67	0.69		
	A	EFV	16.5	10.8	0.66	0.07	0.0310	Potential for interaction; efflux transport of EFV increased
	B	EFV + IVM	8.6	7.7	0.90	0.03		

Drug A Drug whose transport is under investigation

Drug B Drug A plus the interacting drug

ER Efflux ratio

## Discussion

The main aim of this study was to establish the potential interactions between the anthelminthic drugs, IVM and PZQ with ARVs by investigating their transport through CCM. PIs and NNRTIs were selected as they are widely used in management of HIV and may be co-administered with anthelminthics in the mass treatment of helminthic infections and HIV in third world countries because of the geographic overlap of the two diseases. In addition PIs and NNRTIs have both been characterized with regards to substrate specificity of both CYP 450 enzymes and drug transporters [19, 24, 74, 75]. SQV and LPV were selected as prototypes of PIs, while EFV and NVP were prototypes of NNRTIs. The CCM express a wide range of transporters making them suitable for the study of drug-drug interactions, since drug transporters play an integral role in the disposition of drugs and corresponding susceptibility to drug interactions [25, 44, 54–56].

The main findings from our study provide evidence that IVM influences the transport of SQV, LPV and EFV; whereas NVP influences the transport of IVM as illustrated by their transport characteristics along the CCM. IVM significantly inhibited the efflux transport of LPV and SQV; and increased the efflux of EFV. NVP increased the efflux transport of IVM. This raises the possibility of interactions between the drugs involving drug transporters. Drug interactions between ARVs and co-administered drugs may lead to treatment failures and adverse reactions, and understanding of the mechanism of interaction is pivotal for the optimal choice of the highly active antiretroviral therapy (HAART) regimens [76]. Increased efflux of a drug from the cells may cause resistance as the levels become sub-therapeutic [77–79], whereas an inhibition of its efflux may cause enhanced plasma toxicity and subsequent toxicity [80–82].

PZQ did not appear to significantly influence the transport of ARVs and likewise ARVs did not affect the transport of PZQ. SQV and other PIs have been demonstrated to be substrates of efflux transporters P-gp, MRP1 and MRP2 that are expressed by Caco-2 cells [17, 19]. IVM has also been characterized as a substrate of P-gp and has been shown to inhibit P-gp, MRP 1, 2 and 3 [34]. Altered expression of P-gp has been attributed to the neurotoxicity associated with IVM [83]. In an experiment involving collie dogs, the dogs that had a deletion of ABCB-1 gene displayed neurotoxicity when dosed with IVM, whereas normal dogs do not. The authors in this study concluded that P-gp plays a role in effluxing the IVM from the CNS [84]. In a related study on beagle dogs, co-administration of IVM with spinosad, a P-gp inhibitor, has been demonstrated to increase IVM neurotoxicity through the inhibition of P-gp at the blood brain barrier [85].

IVM has been reported to interact with other drugs including doxycycline and albendazole which improve its antiparasitic efficacy [86]. In the control of onchoerciasis, doxycycline was reported to enhance the ivermectin-induced suppression of microfiladermia [87]. Levamisole has been shown to increase the plasma bioavailability of IVM though without necessarily increasing its antiparasitory effects [88]. Previous studies have also reported that ketoconazole substantially increases IVM plasma concentrations in sheep upon co-administration. The authors attributed this to the reversal of P-gp effects [36]. P-gp modulators itraconazole and valspodar have also been shown to increase the concentration of IVM in plasma and gastrointestinal tissues of rats [37].

There was an eightfold inhibition of the efflux transport of LPV in the presence of IVM which could have possibly involved P-gp or other transporters. The transport of LPV and other PIs has been shown to be modulated by efflux transporters P-gp and MRP [12, 89]. Whereas studies have demonstrated that these efflux transporters limit the uptake of ARVs, influence of influx transporters such as Organic anion-transporting polypeptide (OATP) on their modulation and indeed most drugs has not been fully described. Authors from previous studies concluded that an interplay of influx transporter (OATP), efflux transporters (P-gp and MRP) and lipophilicity had implications on the cellular uptake and retention of SQV and LPV in some T-cell lines, CEM, CEM_VBL_ and CEM_1000_ as well as peripheral blood mononuclear cells [90]. In this study, pre-treatment of cells with P-gp and MRP inhibitors, tariquidar (XR9576) for P-gp, and MK571 with frusemide for MRP respectively; followed by subsequent co-incubation with a human OATP substrate, estrone-3-sulphate (E-3-S) resulted in a reduction of the cellular accumulation of SQV and LPV. This may suggest involvement of OATP in the effect of IVM on the disposition of LPV. It is worth noting that LPV (Log Kow =5.94) is more lipophilic than SQV (Log Kow = 2.5), which may contribute to the difference in response to IVM between the two PIs [91–93]. In a related study the authors concluded that IVM may influence the absorption of fexofenadine by interfering with influx and efflux pumps OATP and P-gp [94]. In our study, LPV inhibited the influx transport of IVM, though to a lesser margin (twofold decrease in the efflux ratio, 2.9 to 1.4 [Table 2]). It is therefore evident from these results that there is likelihood of interactions between LPV and IVM, and that these interactions could most likely be influenced by drug transporters (influx and efflux). Further investigations should be carried out to determine the specific transporters responsible for the interactions, and the dosage range that would exhibit these interactions.

NVP increased the efflux transport of IVM, but IVM did not appear to significantly influence the transport of NVP. In a study to investigate the influence of NNRTIs on P-gp activity, NVP significantly reduced the uptake of rhodamine 123, a P-gp substrate into LS180V cells signifying decreased efflux as a result of inhibition of transport [24]. In a related study, the authors concluded that NNRTIs induced P-gp in LS 180 cells [95, 96]. The observed interactions between NVP and IVM may therefore be attributed to the activities of influx and efflux drug transporters. With regards to the interactions between IVM and EFV, there was a marginally significant increase in the efflux ratio of EFV in presence of IVM. EFV has been characterized as a substrate of P-gp and has also been reported to decrease plasma concentrations of co-administered drugs that are metabolized by CYP 450 enzymes without modifying intestinal absorption of co-administered substrates of P-gp [22, 97].

PZQ did not significantly influence the transport of ARVs and likewise ARVs did not significantly affect the transport of PZQ. The presence of PZQ did not alter the transport of SQV, whereas SQV, EFV and NVP did not affect the transport of PZQ. This is consistent with our earlier studies whereby we established that PZQ is neither a substrate nor an inhibitor of P-gp in accumulation experiments involving CEM parental and CEMVBL cells [45]. PZQ has not been conclusively characterized with respect to drug transporters and metabolic enzymes. In a study involving the transport of PZQ and other antiparasitic drugs along Caco-2 cell monolayers, PZQ appeared to be an inhibitor of P-gp without being a substrate, based on inhibition of P-gp mediated [^3^H]-taxol transport in Caco-2 cells [40]. It is however noteworthy that Caco-2 cell lines express several drug transporters, both influx as well as efflux; including metabolic enzymes, and interplay of several factors is therefore possible. Careful interpretation of the results may therefore be necessary before arriving at any conclusions [44, 98, 99]. Ketoconazole, a CYP-450 inhibitor has been reported to double the plasma concentration of PZQ in humans, while rifampicin; an inducer has been reported to dramatically reduce its concentration, and the authors recommended dose adjustment upon co-administration [41, 43]. An increase in Schistosome P-gp levels has also been postulated to confer resistance to PZQ [100].

## Conclusions

This study provides in vitro evidence of potential interactions between IVM, an antihelminthic drug with the ARVs; LPV, SQV and NVP and EFV. Further investigations should be conducted to investigate the possibility of in vivo interactions. From a clinical perspective, the co-administration of IVM with these drugs may require dosage adjustments in order to minimize the incidences of drug-drug interactions. However, this requires corroboration from clinical studies.

## Additional files

Additional file 1: Summary of the methodology. (DOCX 15 kb)

Additional file 2: Transport results. (DOCX 167 kb)

Additional file 3: Apparent permeability (Papp) calculations. (DOCX 23 kb)

Additional file 4: Efflux ratios. (DOCX 13 kb)

Additional file 5: a Impact of SQV on the transport of PZQ along the CCM. b Impact of PZQ on the transport of SQV along the CCM. (ZIP 32 kb)

Additional file 6: a Impact of NVP on the transport of PZQ along the CCM. 2b Impact of EFV on the transport of PZQ along the CCM. (ZIP 30 kb)

Additional file 7: a Impact of SQV on the transport of IVM along the CCM. b Impact of IVM on the transport of SQV along the CCM. (ZIP 29 kb)

Additional file 8: a Impact of NPV on the transport of IVM along the CCM. b Impact of IVM on the transport of NVP along the CCM. (ZIP 29 kb)

Additional file 9: a Impact of LPV on the transport of IVM along the CCM. b Impact of IVM on the transport of LPV along the CCM. (ZIP 29 kb)

Additional file 10: a Impact of EFV on the transport of IVM along the CCM. b Impact of IVM on the transport of EFV along the CCM. (ZIP 28 kb)

## Abbreviations

AIDS: Acquired Immune Deficiency Syndrome
AP: Apical
ARV: Antiretroviral
BL: Basolateral
CCM: Caco-2 cell monolayers
DDIs: Drug-drug interactions
DMEM: Dulbecco's Modified Eagle Medium
DMSO: Dimethyl sulfoxide
EFV: Efavirenz
ER: Efflux ratio
FBS: Fetal bovine serum
HBSS: Hanks' Balanced Salt Solution
HIV: Human Immunodeficiency Virus
HPLC: High Pressure Liquid Chromatography
IVM: Ivermectin
LPV: Lopinavir
MRP: Multidrug resistance-associated protein
NNRTIs: Non-Nucleoside Reverse Transcriptase Inhibitors
NVP: Nevirapine
OATP: Organic anion-transporting polypeptide
P-gp: P-glycoprotein
PIs: Protease inhibitors
PZQ: Praziquantel
SQV: Saquinavir
TEER: Transepithelial resistance
