# Supplementary material for: Drug-transporter mediated interactions between anthelminthic and antiretroviral drugs across the Caco-2 cell monolayers
Source: BMC Pharmacol Toxicol. 2017 May 4;18:20. doi: 10.1186/s40360-017-0129-6 (PMC5415745; doi:10.1186/s40360-017-0129-6)
Supplement: Supplementary file 1 — Summary of the methodology. (DOCX 15 kb) [file 40360_2017_129_MOESM1_ESM.docx]

**i) Summary of the methodology: Impact of SQV on the transport of PZQ along the CCM**

The supplementary files **ii)** to **iv)** serve to illustrate the methods used to calculate the Efflux ratio (ER) from the results taking the *“Impact of SQV on the transport of PZQ along the CCM”* as an example.

**File ii)** – Transport results

The file outlines the transport results. The concentrations for each experiment converted into the amount present as per the 2ml used in picomoles are shown. Plots of the results after the 4hr period are also depicted. The amount transported i.e. from the receiver side are highlighted.

**File iii)** – *P*app calculations

The Table presents the Papp calculations using the results from **File ii**, and the formula

| *P*app (cm/s) | = (dQ/dt) x (1/(AC_O_), where; |
| --- | --- |
| dQ/dt | = Steady-state flux (dpms^-1^ or μmols^-1^) |
| A | = Surface area of the filter (cm^2^) |
| C_O_ | = Initial concentration in the donor chamber (dpm litre^-1^ or μM) |

It also shows the respective Efflux ratio (ER) calculations (Tables C & D), calculated using the formula:

ER = *P*appBA/ *P*appAB

BA = Basolateral to apical

AB = Apical to Basolateral

Please note the following: the efflux ratios are calculated individually for each e.g.

The value for sample 1 highlighted in Table C (0.94) is a product of the value of the first sample in Table B divided by the respective sample in Table A i.e.

094 = 8.46E-05/8.99E-05

A mean and standard deviation of the ER values is then tabulated, 1.14 ± 0.17 in the case of 3600 seconds (Table C).

The means on the right of the *P*app values are the value we reported in the manuscript, and calculating using these *P*app values will yield some slightly variable ER results.

**File iv)** – Efflux ratios (ER)

This is an extension of **File)** to show how we calculated the *P*app and ER values that we presented in **Table 2** of the manuscript.

Please note that the ERs presented are the mean of the individual sample; while the *P*apps are mean as highlighted and also explained in **iii)** above.
