# Supplementary material for: Drug-transporter mediated interactions between anthelminthic and antiretroviral drugs across the Caco-2 cell monolayers
Source: BMC Pharmacol Toxicol. 2017 May 4;18:20. doi: 10.1186/s40360-017-0129-6 (PMC5415745; doi:10.1186/s40360-017-0129-6)
Supplement: Supplementary file 2 — Transport results. (DOCX 167 kb) [file 40360_2017_129_MOESM2_ESM.docx]

**ii)** **Transport results**

|  |  |
| --- | --- |

| **PZQ** | **Apical to basal transport** | | | |  | **Basal to apical transport** | | | |
| --- | --- | --- | --- | --- | --- | --- | --- | --- | --- |
|  | **Amount in 2ml (pmoles)** | |  |  |  | **Amount in 2ml (pmoles)** | |  |  |
| **Time(min)** | **Donor** | **Receiver** | **Mean** | **SDEV** |  | **(μmoles)** | **(pmoles)** | **Mean** | **SDEV** |
| **60** | 22.07 | 16.68 | 15.06 | 1.87 |  | 23.05 | 16.40 | 16.60 | 0.47 |
| **60** | 24.78 | 13.02 |  |  |  | 24.97 | 16.26 |  |  |
| **60** | 23.99 | 15.48 |  |  |  | 21.38 | 17.14 |  |  |
| **120** | 20.34 | 22.40 | 22.74 | 0.31 |  | 21.12 | 19.96 | 20.81 | 0.92 |
| **120** | 20.63 | 22.80 |  |  |  | 22.39 | 21.78 |  |  |
| **120** | 22.73 | 23.02 |  |  |  | 21.38 | 20.68 |  |  |
| **180** | 17.57 | 28.02 | 27.30 | 0.62 |  | 18.34 | 26.18 | 23.94 | 1.98 |
| **180** | 18.31 | 26.92 |  |  |  | 21.65 | 22.42 |  |  |
| **180** | 20.71 | 26.96 |  |  |  | 20.02 | 23.22 |  |  |
| **240** | 16.93 | 30.94 | 32.95 | 2.29 |  | 15.57 | 26.24 | 29.18 | 3.35 |
| **240** | 18.58 | 32.48 |  |  |  | 19.54 | 28.48 |  |  |
| **240** | 19.00 | 35.44 |  |  |  | 17.61 | 32.82 |  |  |
|  |  |  |  |  |  |  |  |  |  |
| **PZQ+SQV** | **Apical to basal transport** | | | |  | **Basal to apical transport** | | | |
|  | **Amount in 2ml (pmoles)** | |  |  |  | **Amount in 2ml (pmoles)** | |  |  |
| **Time(min)** | **Donor** | **Receiver** |  |  |  | **(μmoles)** | **(pmoles)** |  |  |
| **60** | 23.83 | 7.64 | 10.45 | 2.47 |  | 21.23 | 10.64 | 11.67 | 2.63 |
| **60** | 25.29 | 12.28 |  |  |  | 19.14 | 9.70 |  |  |
| **60** | 24.55 | 11.42 |  |  |  | 22.44 | 14.66 |  |  |
| **120** | 21.68 | 21.96 | 21.24 | 1.03 |  | 25.63 | 19.16 | 17.25 | 2.19 |
| **120** | 23.36 | 20.06 |  |  |  | 24.86 | 17.72 |  |  |
| **120** | 24.30 | 21.70 |  |  |  | 21.55 | 14.86 |  |  |
| **180** | 24.23 | 26.76 | 26.22 | 2.12 |  | 20.46 | 24.28 | 25.07 | 0.72 |
| **180** | 21.07 | 28.02 |  |  |  | 18.67 | 25.22 |  |  |
| **180** | 21.17 | 23.88 |  |  |  | 21.67 | 25.70 |  |  |
| **240** | 17.70 | 31.42 | 30.97 | 0.79 |  | 17.74 | 27.60 | 29.78 | 2.56 |
| **240** | 21.69 | 30.06 |  |  |  | 19.91 | 32.60 |  |  |
| **240** | 18.28 | 31.42 |  |  |  | 16.73 | 29.14 |  |  |
