# Supplementary material for: Drug-transporter mediated interactions between anthelminthic and antiretroviral drugs across the Caco-2 cell monolayers
Source: BMC Pharmacol Toxicol. 2017 May 4;18:20. doi: 10.1186/s40360-017-0129-6 (PMC5415745; doi:10.1186/s40360-017-0129-6)
Supplement: Supplementary file 3 — Apparent permeability (Papp) calculations. (DOCX 23 kb) [file 40360_2017_129_MOESM3_ESM.docx]

**01iii) *Papp* calculations** - Apparent permeability coefficient(*P*app) expressed as mean ± s.d of three individual experiments (n=3)

**A) *- P*app PZQ a) Apical to Basal**

| **Time**  **(sec)** | **Receiver**  **(pmoles)** | **Donor**  **(µM)** | **Receiver**  **(pmoles)** | **Receiver**  **(µM)** | **(*dQ*/*dt)*** | **Area**  **(cm^-2^)** | ***Co***  **(μM)** | **(1/(*ACo*)** | ***P*appAB**  **(cm s^-1^)** | **Mean** | **STDEV** |
| --- | --- | --- | --- | --- | --- | --- | --- | --- | --- | --- | --- |
| **3600** | 22.07 | 1.10E-05 | 16.68 | 8.34E-06 | 4.63E-09 | 4.67 | 1.10E-05 | 19404.87 | 8.99E-05 | 7.64E-05 | 1.37E-05 |
| **3600** | 24.78 | 1.24E-05 | 13.02 | 6.51E-06 | 3.62E-09 | 4.67 | 1.24E-05 | 17282.71 | 6.25E-05 |  |  |
| **3600** | 23.99 | 1.20E-05 | 15.48 | 7.74E-06 | 4.30E-09 | 4.67 | 1.20E-05 | 17851.84 | 7.68E-05 |  |  |
| **7200** | 20.34 | 1.02E-05 | 22.40 | 1.12E-05 | 3.11E-09 | 4.67 | 1.02E-05 | 21055.34 | 6.55E-05 | 6.38E-05 | 3.11E-06 |
| **7200** | 20.63 | 1.03E-05 | 22.80 | 1.14E-05 | 3.17E-09 | 4.67 | 1.03E-05 | 20759.36 | 6.57E-05 |  |  |
| **7200** | 22.73 | 1.14E-05 | 23.02 | 1.15E-05 | 3.20E-09 | 4.67 | 1.14E-05 | 18841.42 | 6.02E-05 |  |  |
| **10800** | 17.57 | 8.79E-06 | 28.02 | 1.40E-05 | 2.59E-09 | 4.67 | 8.79E-06 | 24374.82 | 6.32E-05 | 5.77E-05 | 5.83E-06 |
| **10800** | 18.31 | 9.16E-06 | 26.92 | 1.35E-05 | 2.49E-09 | 4.67 | 9.16E-06 | 23389.71 | 5.83E-05 |  |  |
| **10800** | 20.71 | 1.04E-05 | 26.96 | 1.35E-05 | 2.50E-09 | 4.67 | 1.04E-05 | 20679.17 | 5.16E-05 |  |  |
| **14400** | 16.93 | 8.47E-06 | 30.94 | 1.55E-05 | 2.15E-09 | 4.67 | 8.47E-06 | 25296.25 | 5.44E-05 | 5.39E-05 | 1.78E-06 |
| **14400** | 18.58 | 9.29E-06 | 32.48 | 1.62E-05 | 2.26E-09 | 4.67 | 9.29E-06 | 23049.81 | 5.2E-05 |  |  |
| **14400** | 19.00 | 9.50E-06 | 35.44 | 1.77E-05 | 2.46E-09 | 4.67 | 9.50E-06 | 22540.29 | 5.55E-05 |  |  |

**b) Basal to Apical**

| **Basal to Apical** | | | | | | | | | | | |
| --- | --- | --- | --- | --- | --- | --- | --- | --- | --- | --- | --- |
| **Time**  **(sec)** | **Receiver**  **(pmoles)** | **Donor**  **(µM)** | **Receiver**  **(pmoles)** | **Receiver**  **(µM)** | **(*dQ*/*dt)*** | **Area**  **(cm^-2^)** | ***Co*(μM)** | **(1/(*ACo*)** | ***P*appBA**  **(cm s^-1^)** | **Mean** | **STDEV** |
| **3600** | 23.05 | 1.15E-05 | 16.40 | 8.20E-06 | 4.56E-09 | 4.67 | 1.15E-05 | 18579.85 | 8.46E-05 | 8.58E-05 | 9.01E-06 |
| **3600** | 24.97 | 1.25E-05 | 16.26 | 8.13E-06 | 4.52E-09 | 4.67 | 1.25E-05 | 17151.2 | 7.75E-05 |  |  |
| **3600** | 21.38 | 1.07E-05 | 17.14 | 8.57E-06 | 4.76E-09 | 4.67 | 1.07E-05 | 20031.13 | 9.54E-05 |  |  |
| **7200** | 21.12 | 1.06E-05 | 19.96 | 9.98E-06 | 2.77E-09 | 4.67 | 1.06E-05 | 20277.72 | 5.62E-05 | 5.72E-05 | 8.72E-07 |
| **7200** | 22.39 | 1.12E-05 | 21.78 | 1.09E-05 | 3.03E-09 | 4.67 | 1.12E-05 | 19127.54 | 5.79E-05 |  |  |
| **7200** | 21.38 | 1.07E-05 | 20.68 | 1.03E-05 | 2.87E-09 | 4.67 | 1.07E-05 | 20031.13 | 5.75E-05 |  |  |
| **10800** | 18.34 | 9.17E-06 | 26.18 | 1.31E-05 | 2.42E-09 | 4.67 | 9.17E-06 | 23351.45 | 5.66E-05 | 4.79E-05 | 7.94E-06 |
| **10800** | 21.65 | 1.08E-05 | 22.42 | 1.12E-05 | 2.08E-09 | 4.67 | 1.08E-05 | 19781.32 | 4.11E-05 |  |  |
| **10800** | 20.02 | 1.00E-05 | 23.22 | 1.16E-05 | 2.15E-09 | 4.67 | 1.00E-05 | 21391.88 | 4.6E-05 |  |  |
| **14400** | 15.57 | 7.79E-06 | 26.24 | 1.31E-05 | 1.82E-09 | 4.67 | 7.79E-06 | 27505.81 | 5.01E-05 | 4.96E-05 | 6.06E-06 |
| **14400** | 19.54 | 9.77E-06 | 28.48 | 1.42E-05 | 1.98E-09 | 4.67 | 9.77E-06 | 21917.38 | 4.33E-05 |  |  |
| **14400** | 17.61 | 8.81E-06 | 32.82 | 1.64E-05 | 2.28E-09 | 4.67 | 8.81E-06 | 24319.45 | 5.54E-05 |  |  |

**B) - Efflux ratio**

| **PZQ alone** | **Efflux ratio (*P*appBA/*P*appAB)** | | | | |
| --- | --- | --- | --- | --- | --- |
| Time(sec) | 1 | 2 | 3 | Mean | STDEV |
| 3600 | 0.94 | 1.24 | 1.24 | 1.14 | 0.17 |
| 7200 | 0.86 | 0.88 | 0.96 | 0.90 | 0.05 |
| 10800 | 0.90 | 0.70 | 0.89 | 0.83 | 0.11 |
| 14400 | 0.92 | 0.83 | 1.00 | 0.92 | 0.08 |

**C) - *P*app PZQ + SQV a) Apical to Basal**

| **Apical to Basal** | | | | | | | | | | | |
| --- | --- | --- | --- | --- | --- | --- | --- | --- | --- | --- | --- |
| **Time**  **(sec)** | **Donor**  **(pmoles)** | **Donor**  **(µM)** | **Receiver**  **(pmoles)** | **Receiver**  **(µM)** | **(*dQ*/*dt)*** | **Area**  **(cm^-2^)** | ***Co***  **(μM)** | **(1/(*ACo*)** | ***P*appAB**  **(cm s^-1^)** | **Mean** | **STDEV** |
| **3600** | 23.83 | 1.19E-05 | 7.64 | 3.82E-06 | 2.12E-09 | 4.67 | 1.19E-05 | 17971.7 | 3.81E-05 | 5.04E-05 | 1.07E-05 |
| **3600** | 25.29 | 1.26E-05 | 12.28 | 6.14E-06 | 3.41E-09 | 4.67 | 1.26E-05 | 16934.18 | 5.78E-05 |  |  |
| **3600** | 24.55 | 1.23E-05 | 11.42 | 5.71E-06 | 3.17E-09 | 4.67 | 1.23E-05 | 17444.62 | 5.53E-05 |  |  |
| **7200** | 21.68 | 1.08E-05 | 21.96 | 1.10E-05 | 3.05E-09 | 4.67 | 1.08E-05 | 19753.94 | 6.02E-05 | 5.48E-05 | 4.82E-06 |
| **7200** | 23.36 | 1.17E-05 | 20.06 | 1.00E-05 | 2.79E-09 | 4.67 | 1.17E-05 | 18333.28 | 5.11E-05 |  |  |
| **7200** | 24.30 | 1.22E-05 | 21.70 | 1.09E-05 | 3.01E-09 | 4.67 | 1.22E-05 | 17624.1 | 5.31E-05 |  |  |
| **10800** | 24.23 | 1.21E-05 | 26.76 | 1.34E-05 | 2.48E-09 | 4.67 | 1.21E-05 | 17675.01 | 4.38E-05 | 4.71E-05 | 4.91E-06 |
| **10800** | 21.07 | 1.05E-05 | 28.02 | 1.40E-05 | 2.59E-09 | 4.67 | 1.05E-05 | 20325.84 | 5.27E-05 |  |  |
| **10800** | 21.17 | 1.06E-05 | 23.88 | 1.19E-05 | 2.21E-09 | 4.67 | 1.06E-05 | 20229.83 | 4.47E-05 |  |  |
| **14400** | 17.70 | 8.85E-06 | 31.42 | 1.57E-05 | 2.18E-09 | 4.67 | 8.85E-06 | 24195.79 | 5.28E-05 | 4.84E-05 | 6.26E-06 |
| **14400** | 21.69 | 1.08E-05 | 30.06 | 1.50E-05 | 2.09E-09 | 4.67 | 1.08E-05 | 19744.84 | 4.12E-05 |  |  |
| **14400** | 18.28 | 9.14E-06 | 31.42 | 1.57E-05 | 2.18E-09 | 4.67 | 9.14E-06 | 23428.09 | 5.11E-05 |  |  |

**b) Basal to Apical**

|  | **Basal to Apical** | | | | | | | | | | |
| --- | --- | --- | --- | --- | --- | --- | --- | --- | --- | --- | --- |
| **Time (sec)** | **Donor**  **(pmoles)** | **Donor**  **(µM)** | **Receiver**  **(µM)** | **Receiver**  **(pmoles)** | **(*dQ*/*dt)*** | **Area**  **(cm^-2^)** | ***Co***  **(μM)** | **(1/(*ACo*)** | ***P*appBA**  **(cm s^-1^)** | **Mean** | **STDEV** |
| **3600** | 21.23 | 1.06E-05 | 10.64 | 5.32E-06 | 2.96E-09 | 4.67 | 1.06E-05 | 20172.66 | 5.96E-05 | 6.59E-05 | 1.03E-05 |
| **3600** | 19.14 | 9.57E-06 | 9.70 | 4.85E-06 | 2.69E-09 | 4.67 | 9.57E-06 | 22375.42 | 6.03E-05 |  |  |
| **3600** | 22.44 | 1.12E-05 | 14.66 | 7.33E-06 | 4.07E-09 | 4.67 | 1.12E-05 | 19084.92 | 7.77E-05 |  |  |
| **7200** | 25.63 | 1.28E-05 | 19.16 | 9.58E-06 | 2.66E-09 | 4.67 | 1.28E-05 | 16709.54 | 4.45E-05 | 4.26E-05 | 1.74E-06 |
| **7200** | 24.86 | 1.24E-05 | 17.72 | 8.86E-06 | 2.46E-09 | 4.67 | 1.24E-05 | 17227.09 | 4.24E-05 |  |  |
| **7200** | 21.55 | 1.08E-05 | 14.86 | 7.43E-06 | 2.06E-09 | 4.67 | 1.08E-05 | 19873.11 | 4.1E-05 |  |  |
| **10800** | 20.46 | 1.02E-05 | 24.28 | 1.21E-05 | 2.25E-09 | 4.67 | 1.02E-05 | 20931.84 | 4.71E-05 | 4.92E-05 | 3.77E-06 |
| **10800** | 18.67 | 9.34E-06 | 25.22 | 1.26E-05 | 2.34E-09 | 4.67 | 9.34E-06 | 22938.7 | 5.36E-05 |  |  |
| **10800** | 21.67 | 1.08E-05 | 25.70 | 1.29E-05 | 2.38E-09 | 4.67 | 1.08E-05 | 19763.06 | 4.7E-05 |  |  |
| **14400** | 17.74 | 8.87E-06 | 27.60 | 1.38E-05 | 1.92E-09 | 4.67 | 8.87E-06 | 24141.24 | 4.63E-05 | 4.89E-05 | 2.77E-06 |
| **14400** | 19.91 | 9.96E-06 | 32.60 | 1.63E-05 | 2.26E-09 | 4.67 | 9.96E-06 | 21510.07 | 4.87E-05 |  |  |
| **14400** | 16.73 | 8.37E-06 | 29.14 | 1.46E-05 | 2.02E-09 | 4.67 | 8.37E-06 | 25598.66 | 5.18E-05 |  |  |

**D) - Efflux ratio**

| **PZQ + SQV** | **Efflux ratio (*P*appBA/*P*appAB)** | | | | |
| --- | --- | --- | --- | --- | --- |
| **Time (sec)** | **1** | **2** | **3** | **Mean** | **STDEV** |
| **3600** | 1.57 | 0.87 | 1.40 | 1.28 | 0.36 |
| **7200** | 0.74 | 0.83 | 0.77 | 0.78 | 0.05 |
| **10800** | 1.07 | 1.02 | 1.05 | 1.05 | 0.03 |
| **14400** | 0.88 | 1.18 | 1.01 | 1.02 | 0.15 |
