# Supplementary material for: Drug-transporter mediated interactions between anthelminthic and antiretroviral drugs across the Caco-2 cell monolayers
Source: BMC Pharmacol Toxicol. 2017 May 4;18:20. doi: 10.1186/s40360-017-0129-6 (PMC5415745; doi:10.1186/s40360-017-0129-6)
Supplement: Supplementary file 4 — Efflux ratios. (DOCX 13 kb) [file 40360_2017_129_MOESM4_ESM.docx]

**Efflux ratios (ER)**

**i) PZQ alone**

| **PZQ** | ***P*app (cm/s)** | |  |
| --- | --- | --- | --- |
| **Sample** | ***P*appAB** | ***P*appBA** | **ER** |
| 1 | 8.99E-05 | 8.46E-05 | 0.94 |
| 2 | 6.25E-05 | 7.75E-05 | 1.24 |
| 3 | 7.68E-05 | 9.54E-05 | 1.24 |
| **Mean (x10^6^)** | 7.64 | 8.56 | 1.14 |
| **STDEV** |  |  | 0.17 |

**ii) In presence of SQV**

| **PZQ + SQV** | ***P*app (cm/s)** | | |
| --- | --- | --- | --- |
| **Sample** | ***P*appAB** | ***P*appBA** | **ER** |
| 1 | 3.81E-05 | 5.96E-05 | 1.56 |
| 2 | 5.78E-05 | 6.03E-05 | 1.04 |
| 3 | 5.53E-05 | 7.77E-05 | 1.40 |
| **Mean (x10^6^)** | 5.04 | 6.59 | 1.34 |
| **STDEV** |  |  | 0.27 |

**Mean Efflux ratio calculated from each set of results for the whole period**

**i) PZQ alone**

| **PZQ** | ***P*appBA/*P*appAB** | | | | |
| --- | --- | --- | --- | --- | --- |
| **Time(sec)** | **1** | **2** | **3** | **Mean** | **STDEV** |
| **3600** | 0.94 | 1.24 | 1.24 | 1.14 | 0.17 |
| **7200** | 0.86 | 0.88 | 0.96 | 0.90 | 0.05 |
| **10800** | 0.90 | 0.70 | 0.89 | 0.83 | 0.11 |
| **14400** | 0.92 | 0.83 | 1.00 | 0.92 | 0.08 |
|  |  |  |  |  |  |

**ii) In presence of SQV**

| **PZQ + SQV** | ***P*appBA/*P*appAB** | | | | |
| --- | --- | --- | --- | --- | --- |
| **Time(sec)** | **1** | **2** | **3** | **Mean** | **STDEV** |
| **3600** | 1.56 | 1.04 | 1.40 | 1.34 | 0.27 |
| **7200** | 0.74 | 0.83 | 0.77 | 0.78 | 0.05 |
| **10800** | 1.07 | 1.02 | 1.05 | 1.05 | 0.03 |
| **14400** | 0.88 | 1.18 | 1.01 | 1.02 | 0.15 |
