# Supplementary material for: Drug-transporter mediated interactions between anthelminthic and antiretroviral drugs across the Caco-2 cell monolayers
Source: BMC Pharmacol Toxicol. 2017 May 4;18:20. doi: 10.1186/s40360-017-0129-6 (PMC5415745; doi:10.1186/s40360-017-0129-6)
Supplement: Supplementary file 5 — a Impact of SQV on the transport of PZQ along the CCM. b Impact of PZQ on the transport of SQV along the CCM. (ZIP 32 kb) [file 40360_2017_129_MOESM5_ESM.zip › Additional file 1a Impact of SQV on PZQ along the CCMR3.docx]

**Impact of SQV on the transport of PZQ along the CCM**

Apparent permeability coefficient (*P*app) expressed as mean ± S.D of three individual experiments (n=3)

**Cumulative transepithelial transport of PZQ across the CCM alone, and in the presence of SQV**

| **PZQ** | **Apical to basal transport (pmoles)** | | | | |  | **Basal to apical transport (pmoles)** | | | | |
| --- | --- | --- | --- | --- | --- | --- | --- | --- | --- | --- | --- |
| **Time(min)** | **1** | **2** | **3** | **Mean** | **STDEV** |  | **1** | **2** | **3** | **Mean** | **STDEV** |
| **60** | 16.68 | 13.02 | 15.48 | 15.06 | 1.87 |  | 16.40 | 16.26 | 17.14 | 16.60 | 0.47 |
| **120** | 22.40 | 22.80 | 23.02 | 22.74 | 0.31 |  | 19.96 | 21.78 | 20.68 | 20.81 | 0.92 |
| **180** | 28.02 | 26.92 | 26.96 | 27.30 | 0.62 |  | 26.18 | 22.42 | 23.22 | 23.94 | 1.98 |
| **240** | 30.94 | 32.48 | 35.44 | 32.95 | 2.29 |  | 26.24 | 28.48 | 32.82 | 29.18 | 3.35 |
|  |  |  |  |  |  |  |  |  |  |  |  |
| **PZQ + SQV** | **Apical to basal transport (pmoles)** | | | | |  | **Basal to apical transport (pmoles)** | | | | |
| **Time(min)** | **1** | **2** | **3** | **Mean** | **STDEV** |  | **1** | **2** | **3** | **Mean** | **STDEV** |
| **60** | 7.64 | 12.28 | 11.42 | 10.45 | 2.47 |  | 10.64 | 9.70 | 14.66 | 11.67 | 2.63 |
| **120** | 21.96 | 20.06 | 21.70 | 21.24 | 1.03 |  | 19.16 | 17.72 | 14.86 | 17.25 | 2.19 |
| **180** | 26.76 | 28.02 | 23.88 | 26.22 | 2.12 |  | 24.28 | 25.22 | 25.70 | 25.07 | 0.72 |
| **240** | 31.42 | 30.06 | 31.42 | 30.97 | 0.79 |  | 27.60 | 32.60 | 29.14 | 29.78 | 2.56 |

***P*app calculations for the samples after 60min**

|  | **Apical to basal transport** | | | | **Basal to apical transport** | | | | **Efflux ratio** | | | |
| --- | --- | --- | --- | --- | --- | --- | --- | --- | --- | --- | --- | --- |
| **PZQ** | Conc. (pmoles) | | *P*appAB (10^6^ cm/s) | | Conc. (pmoles) | | *P*appBA (10^6^ cm/s) | | **ER** | **Mean** | **STDEV** | ***p***  **value** |
| Sample # | Apical | Basal | *P*app | Mean | Basal | Apical | *P*app | Mean |  |  |  |  |
| 1 | 22.07 | 16.68 | 8.99 | 7.64 | 23.05 | 16.4 | 8.46 | 8.58 | 0.94 | 1.14 | 0.17 | 0.5008 |
| 2 | 24.78 | 13.02 | 6.25 |  | 24.97 | 16.26 | 7.75 |  | 1.24 |  |  |  |
| 3 | 23.99 | 15.48 | 7.68 |  | 21.38 | 17.14 | 9.54 |  | 1.24 |  |  |  |
| **PZQ+SQV** | Apical | Basal | *P*app | Mean | Basal | Apical | *P*app | Mean | **ER** | **Mean** | **STDEV** |  |
| 1 | 23.83 | 7.64 | 3.81 | 5.04 | 21.23 | 10.64 | 5.96 | 6.59 | 1.56 | 1.34 | 0.27 |  |
| 2 | 25.29 | 12.28 | 5.78 |  | 19.14 | 9.70 | 6.03 |  | 1.04 |  |  |  |
| 3 | 24.55 | 11.42 | 5.53 |  | 22.44 | 14.66 | 7.77 |  | 1.40 |  |  |  |
