# Supplementary material for: Drug-transporter mediated interactions between anthelminthic and antiretroviral drugs across the Caco-2 cell monolayers
Source: BMC Pharmacol Toxicol. 2017 May 4;18:20. doi: 10.1186/s40360-017-0129-6 (PMC5415745; doi:10.1186/s40360-017-0129-6)
Supplement: Supplementary file 5 — a Impact of SQV on the transport of PZQ along the CCM. b Impact of PZQ on the transport of SQV along the CCM. (ZIP 32 kb) [file 40360_2017_129_MOESM5_ESM.zip › Additional file 1b Impact of PZQ on SQV along the CCMR3.docx]

**Impact of PZQ on the transport of SQV along the CCM**

Apparent permeability coefficient (*P*app) expressed as mean ± S.D of three individual experiments (n=3)

**Cumulative transepithelial transport of SQV across the CCM alone, and in the presence of PZQ**

| **SQV** | **Apical to basal transport (pmoles)** | | | | |  | **Basal to apical transport (pmoles)** | | | | |
| --- | --- | --- | --- | --- | --- | --- | --- | --- | --- | --- | --- |
| **Time(min)** | **1** | **2** | **3** | **Mean** | **STDEV** |  | **1** | **2** | **3** | **Mean** | **STDEV** |
| **60** | 3.93 | 2.52 | 3.10 | 3.18 | 0.71 |  | 5.98 | 5.60 | 7.29 | 6.29 | 0.89 |
| **120** | 4.59 | 4.09 | 4.29 | 4.32 | 0.26 |  | 10.77 | 11.26 | 12.52 | 11.52 | 0.90 |
| **180** | 5.76 | 5.30 | 6.14 | 5.73 | 0.42 |  | 16.14 | 16.78 | 15.35 | 16.09 | 0.71 |
| **240** | 6.59 | 5.67 | 6.93 | 6.40 | 0.65 |  | 18.19 | 20.22 | 23.27 | 20.56 | 2.56 |
|  |  |  |  |  |  |  |  |  |  |  |  |
| **SQV + PZQ** | **Apical to basal transport (pmoles)** | | | | |  | **Basal to apical transport (pmoles)** | | | | |
| **Time(min)** | **1** | **2** | **3** | **Mean** | **STDEV** |  | **1** | **2** | **3** | **Mean** | **STDEV** |
| **60** | 3.78 | 5.47 | 4.87 | 4.71 | 0.86 |  | 11.43 | 8.40 | 9.62 | 9.82 | 1.52 |
| **120** | 4.61 | 5.42 | 5.55 | 5.19 | 0.51 |  | 17.83 | 12.79 | 15.63 | 15.42 | 2.53 |
| **180** | 4.68 | 5.54 | 5.48 | 5.24 | 0.48 |  | 21.13 | 19.51 | 23.31 | 21.32 | 1.91 |
| **240** | 5.67 | 5.60 | 6.53 | 5.93 | 0.52 |  | 29.11 | 22.98 | 27.62 | 26.57 | 3.20 |

***P*app calculations for the samples after 60min**

|  | **Apical to basal transport** | | | | **Basal to apical transport** | | | | **Efflux ratio** | | | |
| --- | --- | --- | --- | --- | --- | --- | --- | --- | --- | --- | --- | --- |
| **SQV** | Conc. (pmoles) | | *P*appAB (10^6^ cm/s) | | Conc. (pmoles) | | *P*appBA (10^6^ cm/s) | | **ER** | **Mean** | **STDEV** | ***p***  **value** |
| Sample # | Apical | Basal | *P*app | Mean | Basal | Apical | *P*app | Mean |  |  |  |  |
| 1 | 40.19 | 3.93 | 11.63 | 6.56 | 49.49 | 3.78 | 9.08 | 10.75 | 1.58 | 3.55 | 1.72 | 0.6796 |
| 2 | 40.98 | 2.52 | 3.66 |  | 52.97 | 5.47 | 12.28 |  | 4.41 |  |  |  |
| 3 | 42.05 | 3.10 | 4.38 |  | 53.30 | 4.87 | 10.88 |  | 4.67 |  |  |  |
| **SQV+PZQ** | Apical | Basal | *P*app | Mean | Basal | Apical | *P*app | Mean | **ER** | **Mean** | **STDEV** |  |
| 1 | 38.83 | 5.98 | 18.33 | 18.31 | 33.63 | 11.43 | 40.42 | 27.03 | 4.45 | 2.65 | 1.57 |  |
| 2 | 41.31 | 5.60 | 16.13 |  | 42.80 | 8.40 | 23.34 |  | 1.90 |  |  |  |
| 3 | 42.34 | 7.29 | 20.48 |  | 66.04 | 9.62 | 17.33 |  | 1.59 |  |  |  |
