# Supplementary material for: Drug-transporter mediated interactions between anthelminthic and antiretroviral drugs across the Caco-2 cell monolayers
Source: BMC Pharmacol Toxicol. 2017 May 4;18:20. doi: 10.1186/s40360-017-0129-6 (PMC5415745; doi:10.1186/s40360-017-0129-6)
Supplement: Supplementary file 6 — a Impact of NVP on the transport of PZQ along the CCM. 2b Impact of EFV on the transport of PZQ along the CCM. (ZIP 30 kb) [file 40360_2017_129_MOESM6_ESM.zip › Additional file 2a Impact of NVP on PZQ transport the CCMR3.docx]

**Impact of NVP on the transport of PZQ along the CCM**

Apparent permeability coefficient (*P*app) expressed as mean ± S.D of three individual experiments (n=3)

**Cumulative transepithelial transport of PZQ across the CCM alone, and in the presence of NVP**

| **PZQ** | **Apical to basal transport (pmoles)** | | | | |  | **Basal to apical transport (pmoles)** | | | | |
| --- | --- | --- | --- | --- | --- | --- | --- | --- | --- | --- | --- |
| **Time(min)** | **1** | **2** | **3** | **Mean** | **STDEV** |  | **1** | **2** | **3** | **Mean** | **STDEV** |
| **60** | 22.50 | 22.28 | 25.50 | 23.43 | 1.80 |  | 23.94 | 25.98 | 25.28 | 25.07 | 1.04 |
| **120** | 34.88 | 39.18 | 45.74 | 39.93 | 5.47 |  | 49.34 | 45.84 | 54.66 | 49.95 | 4.44 |
| **180** | 46.84 | 53.00 | 50.62 | 50.15 | 3.11 |  | 53.14 | 59.00 | 51.94 | 54.69 | 3.78 |
| **240** | 63.58 | 68.96 | 74.76 | 69.10 | 5.59 |  | 65.74 | 78.78 | 75.20 | 73.24 | 6.74 |
|  |  |  |  |  |  |  |  |  |  |  |  |
| **PZQ + NVP** | **Apical to basal transport (pmoles)** | | | | |  | **Basal to apical transport (pmoles)** | | | | |
| **Time(min)** | **1** | **2** | **3** | **Mean** | **STDEV** |  | **1** | **2** | **3** | **Mean** | **STDEV** |
| **60** | 31.72 | 28.33 | 27.56 | 29.20 | 2.21 |  | 27.42 | 25.73 | 23.74 | 25.63 | 1.84 |
| **120** | 39.46 | 33.96 | 39.74 | 37.72 | 3.26 |  | 37.72 | 40.31 | 43.83 | 40.62 | 3.07 |
| **180** | 47.46 | 50.75 | 53.28 | 50.50 | 2.92 |  | 44.02 | 59.19 | 59.29 | 54.17 | 8.79 |
| **240** | 55.41 | 59.86 | 67.02 | 60.76 | 5.86 |  | 69.52 | 63.93 | 65.83 | 66.43 | 2.84 |

***P*app calculations for the samples after 60min**

|  | **Apical to basal transport** | | | | **Basal to apical transport** | | | | **Efflux ratio** | | | |
| --- | --- | --- | --- | --- | --- | --- | --- | --- | --- | --- | --- | --- |
| **PZQ** | Conc. (pmoles) | | *P*appAB (10^6^ cm/s) | | Conc. (pmoles) | | *P*appBA (10^6^ cm/s) | | **ER** | **Mean** | **STDEV** | ***p***  **value** |
| Sample # | Apical | Basal | *P*app | Mean | Basal | Apical | *P*app | Mean |  |  |  |  |
| 1 | 52.45 | 22.50 | 25.52 | 28.10 | 44.74 | 23.94 | 31.83 | 28.63 | 1.25 | 1.03 | 0.19 | 0.0964 |
| 2 | 43.25 | 22.28 | 30.64 |  | 53.31 | 25.98 | 28.99 |  | 0.95 |  |  |  |
| 3 | 53.90 | 25.50 | 28.14 |  | 59.95 | 25.28 | 25.08 |  | 0.89 |  |  |  |
| **PZQ+NVP** | Apical | Basal | *P*app | Mean | Basal | Apical | *P*app | Mean | **ER** | **Mean** | **STDEV** |  |
| 1 | 46.10 | 31.72 | 40.93 | 37.16 | 44.09 | 27.42 | 36.99 | 30.51 | 0.90 | 0.82 | 0.08 |  |
| 2 | 45.56 | 28.33 | 36.99 |  | 54.85 | 25.73 | 27.90 |  | 0.75 |  |  |  |
| 3 | 48.84 | 27.56 | 33.56 |  | 53.04 | 23.74 | 26.63 |  | 0.79 |  |  |  |
