# Supplementary material for: Drug-transporter mediated interactions between anthelminthic and antiretroviral drugs across the Caco-2 cell monolayers
Source: BMC Pharmacol Toxicol. 2017 May 4;18:20. doi: 10.1186/s40360-017-0129-6 (PMC5415745; doi:10.1186/s40360-017-0129-6)
Supplement: Supplementary file 6 — a Impact of NVP on the transport of PZQ along the CCM. 2b Impact of EFV on the transport of PZQ along the CCM. (ZIP 30 kb) [file 40360_2017_129_MOESM6_ESM.zip › Additional file 2b Impact of EFV on PZQ along the CCMR3.docx]

**Impact of EFV on the transport of PZQ along the CCM**

Apparent permeability coefficient (*P*app) expressed as mean ± S.D of three individual experiments (n=3)

**Cumulative transepithelial transport of PZQ across the CCM alone, and in the presence of EFV**

| **PZQ** | **Apical to basal transport (pmoles)** | | | | |  | **Basal to apical transport (pmoles)** | | | | |
| --- | --- | --- | --- | --- | --- | --- | --- | --- | --- | --- | --- |
| **Time(min)** | **1** | **2** | **3** | **Mean** | **STDEV** |  | **1** | **2** | **3** | **Mean** | **STDEV** |
| **60** | 33.50 | 22.28 | 39.18 | 31.65 | 8.60 |  | 25.94 | 27.98 | 25.28 | 26.40 | 1.41 |
| **120** | 45.74 | 32.50 | 34.88 | 37.71 | 7.06 |  | 49.34 | 35.84 | 54.66 | 46.61 | 9.70 |
| **180** | 46.84 | 53.00 | 63.58 | 54.47 | 8.47 |  | 53.14 | 51.00 | 71.74 | 58.63 | 11.41 |
| **240** | 50.62 | 68.96 | 94.76 | 71.45 | 22.17 |  | 51.94 | 74.78 | 75.20 | 67.31 | 13.31 |
|  |  |  |  |  |  |  |  |  |  |  |  |
| **PZQ + EFV** | **Apical to basal transport (pmoles)** | | | | |  | **Basal to apical transport (pmoles)** | | | | |
| **Time(min)** | **1** | **2** | **3** | **Mean** | **STDEV** |  | **1** | **2** | **3** | **Mean** | **STDEV** |
| **60** | 31.64 | 28.70 | 21.84 | 27.39 | 5.03 |  | 29.86 | 29.70 | 32.96 | 30.84 | 1.84 |
| **120** | 40.82 | 42.86 | 47.36 | 43.68 | 3.35 |  | 42.36 | 39.06 | 55.00 | 45.47 | 8.41 |
| **180** | 49.20 | 53.96 | 42.00 | 48.39 | 6.02 |  | 49.82 | 46.46 | 50.00 | 48.76 | 1.99 |
| **240** | 49.22 | 49.76 | 79.32 | 59.43 | 17.22 |  | 51.88 | 48.36 | 50.26 | 50.17 | 1.76 |

***P*app calculations for the samples after 60min**

|  | **Apical to basal transport** | | | | **Basal to apical transport** | | | | **Efflux ratio** | | | |
| --- | --- | --- | --- | --- | --- | --- | --- | --- | --- | --- | --- | --- |
| **PZQ** | Conc. (pmoles) | | *P*appAB (10^6^ cm/s) | | Conc. (pmoles) | | *P*appBA (10^6^ cm/s) | | **ER** | **Mean** | **STDEV** | ***p***  **value** |
| Sample # | Apical | Basal | *P*app | Mean | Basal | Apical | *P*app | Mean |  |  |  |  |
| 1 | 52.45 | 33.50 | 37.99 | 35.03 | 44.74 | 25.94 | 34.49 | 30.26 | 0.91 | 0.87 | 0.17 | 0.4676 |
| 2 | 43.25 | 22.28 | 30.64 |  | 53.31 | 27.98 | 31.22 |  | 1.02 |  |  |  |
| 3 | 63.90 | 39.18 | 36.47 |  | 59.95 | 25.28 | 25.08 |  | 0.69 |  |  |  |
| **PZQ+EFV** | Apical | Basal | *P*app | Mean | Basal | Apical | *P*app | Mean | **ER** | **Mean** | **STDEV** |  |
| 1 | 43.01 | 31.64 | 43.76 | 35.07 | 47.85 | 29.86 | 37.12 | 39.15 | 0.85 | 1.19 | 0.48 |  |
| 2 | 47.76 | 28.70 | 35.74 |  | 49.66 | 29.70 | 35.57 |  | 1.00 |  |  |  |
| 3 | 50.52 | 21.84 | 25.71 |  | 43.79 | 32.96 | 44.77 |  | 1.74 |  |  |  |
