# Supplementary material for: Drug-transporter mediated interactions between anthelminthic and antiretroviral drugs across the Caco-2 cell monolayers
Source: BMC Pharmacol Toxicol. 2017 May 4;18:20. doi: 10.1186/s40360-017-0129-6 (PMC5415745; doi:10.1186/s40360-017-0129-6)
Supplement: Supplementary file 7 — a Impact of SQV on the transport of IVM along the CCM. b Impact of IVM on the transport of SQV along the CCM. (ZIP 29 kb) [file 40360_2017_129_MOESM7_ESM.zip › Additional file 3a Impact of SQV on IVM along the CCMR3.docx]

**Impact of SQV on the transport of IVM along the CCM**

Apparent permeability coefficient (*P*app) expressed as mean ± S.D of three individual experiments (n=3)

**Cumulative transepithelial transport of IVM across the CCM alone, and in the presence of SQV**

| **IVM** | **Apical to basal transport (fmoles)** | | | | |  | **Basal to apical transport (fmoles)** | | | | |
| --- | --- | --- | --- | --- | --- | --- | --- | --- | --- | --- | --- |
| **Time(min)** | **1** | **2** | **3** | **Mean** | **STDEV** |  | **1** | **2** | **3** | **Mean** | **STDEV** |
| **60** | 15.84 | 11.06 | 7.45 | 11.45 | 4.21 |  | 13.53 | 12.29 | 9.26 | 11.69 | 2.20 |
| **120** | 14.08 | 21.31 | 12.37 | 15.92 | 4.75 |  | 21.06 | 15.84 | 16.78 | 17.90 | 2.78 |
| **180** | 23.29 | 18.34 | 20.63 | 20.75 | 2.48 |  | 25.47 | 26.26 | 25.52 | 25.75 | 0.45 |
| **240** | 21.18 | 30.03 | 25.60 | 25.60 | 4.42 |  | 45.23 | 24.14 | 36.53 | 35.30 | 10.60 |
|  |  |  |  |  |  |  |  |  |  |  |  |
| **IVM + SQV** | **Apical to basal transport (fmoles)** | | | | |  | **Basal to apical transport (fmoles)** | | | | |
| **Time(min)** | **1** | **2** | **3** | **Mean** | **STDEV** |  | **1** | **2** | **3** | **Mean** | **STDEV** |
| **60** | 14.83 | 13.57 | 11.46 | 13.29 | 1.70 |  | 14.41 | 15.36 | 18.90 | 16.23 | 2.37 |
| **120** | 32.46 | 19.38 | 21.83 | 24.56 | 6.95 |  | 30.99 | 28.46 | 35.96 | 31.80 | 3.81 |
| **180** | 33.51 | 34.76 | 31.64 | 33.30 | 1.57 |  | 40.46 | 39.99 | 33.11 | 37.86 | 4.11 |
| **240** | 43.06 | 30.67 | 40.12 | 37.95 | 6.47 |  | 45.05 | 44.34 | 52.14 | 47.18 | 4.32 |

***P*app calculations for the samples after 60min**

|  | **Apical to basal transport** | | | | **Basal to apical transport** | | | | **Efflux ratio** | | | |
| --- | --- | --- | --- | --- | --- | --- | --- | --- | --- | --- | --- | --- |
| **IVM** | Conc. (fmoles) | | *P*appAB (10^6^ cm/s) | | Conc. (fmoles) | | *P*appBA (10^6^ cm/s) | | **ER** | **Mean** | **STDEV** | ***p***  **value** |
| Sample # | Apical | Basal | *P*app | Mean | Basal | Apical | *P*app | Mean |  |  |  |  |
| 1 | 175.49 | 15.84 | 10.74 | 7.41 | 218.64 | 13.53 | 7.36 | 5.92 | 0.70 | 0.86 | 0.18 | 0.2007 |
| 2 | 183.22 | 11.06 | 7.18 |  | 250.17 | 12.29 | 5.84 |  | 0.82 |  |  |  |
| 3 | 205.30 | 7.45 | 4.31 |  | 242.33 | 9.26 | 4.54 |  | 1.05 |  |  |  |
| **IVM+SQV** | Apical | Basal | *P*app | Mean | Basal | Apical | *P*app | Mean | **ER** | **Mean** | **STDEV** |  |
| 1 | 242.27 | 14.83 | 7.28 | 6.42 | 242.43 | 14.41 | 7.07 | 6.33 | 0.97 | 0.99 | 0.16 |  |
| 2 | 255.62 | 13.57 | 6.32 |  | 346.74 | 15.36 | 5.27 |  | 0.84 |  |  |  |
| 3 | 241.14 | 11.46 | 5.65 |  | 337.71 | 18.90 | 6.66 |  | 1.17 |  |  |  |
