# Supplementary material for: Drug-transporter mediated interactions between anthelminthic and antiretroviral drugs across the Caco-2 cell monolayers
Source: BMC Pharmacol Toxicol. 2017 May 4;18:20. doi: 10.1186/s40360-017-0129-6 (PMC5415745; doi:10.1186/s40360-017-0129-6)
Supplement: Supplementary file 7 — a Impact of SQV on the transport of IVM along the CCM. b Impact of IVM on the transport of SQV along the CCM. (ZIP 29 kb) [file 40360_2017_129_MOESM7_ESM.zip › Additional file 3b Impact of IVM on SQV along the CCMR3.docx]

**Impact of IVM on the transport of SQV along the CCM**

Apparent permeability coefficient (*P*app) expressed as mean ± S.D of three individual experiments (n=3)

**Cumulative transepithelial transport of SQV across the CCM alone, and in the presence of IVM**

| **SQV** | **Apical to basal transport (pmoles)** | | | | |  | **Basal to apical transport (pmoles)** | | | | |
| --- | --- | --- | --- | --- | --- | --- | --- | --- | --- | --- | --- |
| **Time(min)** | **1** | **2** | **3** | **Mean** | **STDEV** |  | **1** | **2** | **3** | **Mean** | **STDEV** |
| **60** | 2.74 | 2.54 | 3.32 | 2.87 | 0.41 |  | 8.18 | 6.66 | 9.42 | 8.09 | 1.38 |
| **120** | 5.56 | 4.48 | 6.52 | 5.52 | 1.02 |  | 13.98 | 11.58 | 17.26 | 14.27 | 2.85 |
| **180** | 7.42 | 7.24 | 8.40 | 7.69 | 0.62 |  | 18.6 | 17.14 | 18.78 | 18.17 | 0.90 |
| **240** | 9.18 | 8.8 | 11.24 | 9.74 | 1.31 |  | 21.42 | 22.46 | 26.66 | 23.51 | 2.77 |
|  |  |  |  |  |  |  |  |  |  |  |  |
| **SQV + IVM** | **Apical to basal transport (pmoles)** | | | | |  | **Basal to apical transport (pmoles)** | | | | |
| **Time(min)** | **1** | **2** | **3** | **Mean** | **STDEV** |  | **1** | **2** | **3** | **Mean** | **STDEV** |
| **60** | 2.18 | 2 | 2.42 | 2.20 | 0.21 |  | 2.66 | 2.58 | 3.08 | 2.77 | 0.27 |
| **120** | 4.70 | 3.86 | 4.88 | 4.48 | 0.54 |  | 4.82 | 4.94 | 6.2 | 5.32 | 0.76 |
| **180** | 5.46 | 4.96 | 6.92 | 5.78 | 1.02 |  | 6.68 | 6.60 | 8.66 | 7.31 | 1.17 |
| **240** | 7.48 | 6.84 | 7.6 | 7.31 | 0.41 |  | 8.98 | 6.74 | 9.9 | 8.54 | 1.63 |

***P*app calculations for the samples after 60min**

|  | **Apical to basal transport** | | | | **Basal to apical transport** | | | | **Efflux ratio** | | | |
| --- | --- | --- | --- | --- | --- | --- | --- | --- | --- | --- | --- | --- |
| **SQV** | Conc. (pmoles) | | *P*appAB (10^6^ cm/s) | | Conc. (pmoles) | | *P*appBA (10^6^ cm/s) | | **ER** | **Mean** | **STDEV** | ***p***  **value** |
| Sample # | Apical | Basal | *P*app | Mean | Basal | Apical | *P*app | Mean |  |  |  |  |
| 1 | 23.25 | 2.74 | 7.01 | 6.91 | 19.35 | 8.18 | 25.15 | 21.13 | 3.59 | 3.05 | 0.46 | 0.0328 |
| 2 | 22.93 | 2.54 | 6.59 |  | 21.54 | 6.66 | 18.39 |  | 2.79 |  |  |  |
| 3 | 27.69 | 3.32 | 7.13 |  | 28.21 | 9.42 | 19.86 |  | 2.79 |  |  |  |
| **SQV+IVM** | Apical | Basal | *P*app | Mean | Basal | Apical | *P*app | Mean | **ER** | **Mean** | **STDEV** |  |
| 1 | 19.84 | 2.18 | 6.54 | 6.20 | 21.81 | 2.66 | 7.25 | 7.67 | 1.11 | 1.24 | 0.14 |  |
| 2 | 19.61 | 2.00 | 6.07 |  | 18.23 | 2.58 | 8.42 |  | 1.39 |  |  |  |
| 3 | 24.04 | 2.42 | 5.99 |  | 24.93 | 3.08 | 7.35 |  | 1.23 |  |  |  |
