# Supplementary material for: Drug-transporter mediated interactions between anthelminthic and antiretroviral drugs across the Caco-2 cell monolayers
Source: BMC Pharmacol Toxicol. 2017 May 4;18:20. doi: 10.1186/s40360-017-0129-6 (PMC5415745; doi:10.1186/s40360-017-0129-6)
Supplement: Supplementary file 8 — a Impact of NPV on the transport of IVM along the CCM. b Impact of IVM on the transport of NVP along the CCM. (ZIP 29 kb) [file 40360_2017_129_MOESM8_ESM.zip › Additional file 4a Impact of NPV on IVM along the CCMR3.docx]

**Impact of NVP on the transport of IVM along the CCM**

Apparent permeability coefficient (*P*app) expressed as mean ± S.D of three individual experiments (n=3)

**Cumulative transepithelial transport of [^3^H] IVM across the CCM alone, and in the presence of NVP**

| **IVM** | **Apical to basal transport (fmoles)** | | | | |  | **Basal to apical transport (fmoles)** | | | | |
| --- | --- | --- | --- | --- | --- | --- | --- | --- | --- | --- | --- |
| **Time(min)** | **1** | **2** | **3** | **Mean** | **STDEV** |  | **1** | **2** | **3** | **Mean** | **STDEV** |
| **60** | 14.35 | 14.86 | 15.44 | 14.88 | 0.54 |  | 13.56 | 12.86 | 12.68 | 13.03 | 0.47 |
| **120** | 20.80 | 20.34 | 21.75 | 20.96 | 0.72 |  | 17.34 | 18.56 | 18.52 | 18.14 | 0.69 |
| **180** | 25.72 | 23.95 | 25.43 | 25.03 | 0.95 |  | 17.07 | 23.80 | 31.79 | 24.22 | 7.37 |
| **240** | 25.16 | 26.38 | 23.06 | 24.86 | 1.68 |  | 22.70 | 28.67 | 34.20 | 28.52 | 5.76 |
|  |  |  |  |  |  |  |  |  |  |  |  |
| **IVM + NVP** | **Apical to basal transport (fmoles)** | | | | |  | **Basal to apical transport (fmoles)** | | | | |
| **Time(min)** | **1** | **2** | **3** | **Mean** | **STDEV** |  | **1** | **2** | **3** | **Mean** | **STDEV** |
| **60** | 5.33 | 5.24 | 5.26 | 5.27 | 0.05 |  | 9.46 | 9.63 | 8.92 | 9.34 | 0.37 |
| **120** | 7.39 | 7.43 | 7.02 | 7.28 | 0.23 |  | 11.37 | 13.98 | 13.12 | 12.82 | 1.33 |
| **180** | 10.92 | 9.14 | 10.48 | 10.18 | 0.93 |  | 11.88 | 11.02 | 16.64 | 13.18 | 3.03 |
| **240** | 10.99 | 7.40 | 8.77 | 9.05 | 1.81 |  | 17.17 | 18.98 | 19.50 | 18.55 | 1.22 |

***P*app calculations for the samples after 60min**

|  | **Apical to basal transport** | | | | **Basal to apical transport** | | | | **Efflux ratio** | | | |
| --- | --- | --- | --- | --- | --- | --- | --- | --- | --- | --- | --- | --- |
| **IVM** | Conc. (fmoles) | | *P*appAB (10^6^ cm/s) | | Conc. (fmoles) | | *P*appBA (10^6^ cm/s) | | **ER** | **Mean** | **STDEV** | ***p***  **value** |
| Sample # | Apical | Basal | *P*app | Mean | Basal | Apical | *P*app | Mean |  |  |  |  |
| 1 | 101.63 | 14.35 | 16.80 | 14.06 | 110.67 | 13.56 | 14.58 | 11.32 | 0.87 | 0.80 | 0.07 | 0.0094 |
| 2 | 106.60 | 14.86 | 13.65 |  | 126.53 | 12.86 | 9.96 |  | 0.73 |  |  |  |
| 3 | 109.59 | 15.44 | 11.73 |  | 111.99 | 12.68 | 9.43 |  | 0.80 |  |  |  |
| **IVM+NVP** | Apical | Basal | *P*app | Mean | Basal | Apical | *P*app | Mean | **ER** | **Mean** | **STDEV** |  |
| 1 | 108.60 | 5.33 | 5.84 | 4.84 | 107.05 | 9.46 | 10.51 | 8.57 | 1.80 | 1.76 | 0.13 |  |
| 2 | 113.58 | 5.24 | 4.52 |  | 111.58 | 9.63 | 8.46 |  | 1.87 |  |  |  |
| 3 | 105.08 | 5.26 | 4.17 |  | 110.25 | 8.92 | 6.74 |  | 1.62 |  |  |  |
