# Supplementary material for: Drug-transporter mediated interactions between anthelminthic and antiretroviral drugs across the Caco-2 cell monolayers
Source: BMC Pharmacol Toxicol. 2017 May 4;18:20. doi: 10.1186/s40360-017-0129-6 (PMC5415745; doi:10.1186/s40360-017-0129-6)
Supplement: Supplementary file 8 — a Impact of NPV on the transport of IVM along the CCM. b Impact of IVM on the transport of NVP along the CCM. (ZIP 29 kb) [file 40360_2017_129_MOESM8_ESM.zip › Additional file 4b Impact of IVM on NVP along the CCMR3.docx]

**Impact of IVM on the transport of NVP along the CCM**

Apparent permeability coefficient (*P*app) expressed as mean ± S.D of three individual experiments (n=3)

**Cumulative transepithelial transport of [^3^H] NPV across the CCM alone, and in the presence of IVM**

| **NVP** | **Apical to basal transport (pmoles)** | | | | |  | **Basal to apical transport (pmoles)** | | | | |
| --- | --- | --- | --- | --- | --- | --- | --- | --- | --- | --- | --- |
| **Time(min)** | **1** | **2** | **3** | **Mean** | **STDEV** |  | **1** | **2** | **3** | **Mean** | **STDEV** |
| **60** | 78.8 | 80.8 | 79.4 | 79.68 | 1.03 |  | 59.7 | 67.9 | 77.2 | 68.27 | 8.73 |
| **120** | 111.5 | 123.2 | 109.9 | 114.88 | 7.28 |  | 81.9 | 91.5 | 105.1 | 92.84 | 11.68 |
| **180** | 116.3 | 120.3 | 122.0 | 119.53 | 2.94 |  | 92.9 | 107.5 | 118.9 | 106.41 | 13.02 |
| **240** | 123.9 | 122.5 | 120.2 | 122.19 | 1.89 |  | 98.8 | 112.2 | 132.2 | 114.39 | 16.83 |
|  |  |  |  |  |  |  |  |  |  |  |  |
| **NVP + IVM** | **Apical to basal transport (pmoles)** | | | | |  | **Basal to apical transport (pmoles)** | | | | |
| **Time(min)** | **1** | **2** | **3** | **Mean** | **STDEV** |  | **1** | **2** | **3** | **Mean** | **STDEV** |
| **60** | 83.9 | 59.4 | 58.1 | 67.13 | 14.54 |  | 64.6 | 62.9 | 78.9 | 68.84 | 8.78 |
| **120** | 113.0 | 100.9 | 102.5 | 105.44 | 6.58 |  | 94.3 | 94.7 | 113.3 | 100.76 | 10.86 |
| **180** | 122.5 | 108.4 | 117.0 | 115.95 | 7.12 |  | 104.8 | 108.1 | 120.3 | 111.09 | 8.14 |
| **240** | 126.8 | 109.8 | 122.7 | 119.77 | 8.91 |  | 110.3 | 117.2 | 131.9 | 119.79 | 11.01 |

***P*app calculations for the samples after 60min**

|  | **Apical to basal transport** | | | | **Basal to apical transport** | | | | **Efflux ratio** | | | |
| --- | --- | --- | --- | --- | --- | --- | --- | --- | --- | --- | --- | --- |
| **NVP** | Conc. (pmoles) | | *P*appAB (10^6^ cm/s) | | Conc. (pmoles) | | *P*appBA (10^6^ cm/s) | | **ER** | **Mean** | **STDEV** | ***p***  **value** |
| Sample # | Apical | Basal | *P*app | Mean | Basal | Apical | *P*app | Mean |  |  |  |  |
| 1 | 177.1 | 78.8 | 52.94 | 42.72 | 129.9 | 59.7 | 54.72 | 39.50 | 1.03 | 0.91 | 0.12 | 0.6891 |
| 2 | 196.6 | 80.8 | 40.28 |  | 207.3 | 67.9 | 32.08 |  | 0.80 |  |  |  |
| 3 | 189.3 | 79.4 | 34.93 |  | 202.9 | 77.2 | 31.69 |  | 0.91 |  |  |  |
| **NVP + IVM** | Apical | Basal | *P*app | Mean | Basal | Apical | *P*app | Mean | **ER** | **Mean** | **STDEV** |  |
| 1 | 157.9 | 83.9 | 63.22 | 39.38 | 146.0 | 64.6 | 52.67 | 36.56 | 0.83 | 0.98 | 0.17 |  |
| 2 | 194.8 | 59.4 | 29.88 |  | 222.4 | 62.9 | 27.74 |  | 0.93 |  |  |  |
| 3 | 193.1 | 58.1 | 25.05 |  | 224.6 | 78.9 | 29.27 |  | 1.17 |  |  |  |
