# Supplementary material for: Drug-transporter mediated interactions between anthelminthic and antiretroviral drugs across the Caco-2 cell monolayers
Source: BMC Pharmacol Toxicol. 2017 May 4;18:20. doi: 10.1186/s40360-017-0129-6 (PMC5415745; doi:10.1186/s40360-017-0129-6)
Supplement: Supplementary file 9 — a Impact of LPV on the transport of IVM along the CCM. b Impact of IVM on the transport of LPV along the CCM. (ZIP 29 kb) [file 40360_2017_129_MOESM9_ESM.zip › Additional file 5a Impact of LPV on IVM along the CCMR3.docx]

**Impact of LPV on the transport of IVM along the CCM**

Apparent permeability coefficient (*P*app) expressed as mean ± S.D of three individual experiments (n=3)

**Cumulative transepithelial transport of [^14^C] LPV across the CCM alone, and in the presence of IVM**

| **IVM** | **Apical to basal transport (pmoles)** | | | | |  | **Basal to apical transport (pmoles)** | | | | |
| --- | --- | --- | --- | --- | --- | --- | --- | --- | --- | --- | --- |
| **Time(min)** | **1** | **2** | **3** | **Mean** | **STDEV** |  | **1** | **2** | **3** | **Mean** | **STDDEV** |
| **60** | 8.30 | 7.46 | 8.35 | 8.04 | 0.50 |  | 16.19 | 15.75 | 21.16 | 17.70 | 3.01 |
| **120** | 17.04 | 10.98 | 9.22 | 12.41 | 4.10 |  | 21.40 | 27.21 | 31.37 | 26.66 | 5.01 |
| **180** | 20.27 | 13.19 | 16.36 | 16.61 | 3.55 |  | 25.52 | 26.81 | 40.70 | 31.01 | 8.42 |
| **240** | 16.26 | 22.17 | 13.75 | 17.39 | 4.33 |  | 32.09 | 35.20 | 45.80 | 37.70 | 7.19 |
|  |  |  |  |  |  |  |  |  |  |  |  |
| **IVM + EFV** | **Apical to basal transport (pmoles)** | | | | |  | **Basal to apical transport (pmoles)** | | | | |
| **Time(min)** | **1** | **2** | **3** | **Mean** | **STDEV** |  | **1** | **2** | **3** | **Mean** | **STDEV** |
| **60** | 14.84 | 9.61 | 9.16 | 11.20 | 3.16 |  | 12.20 | 15.87 | 16.19 | 14.75 | 2.22 |
| **120** | 27.40 | 13.14 | 18.31 | 19.62 | 7.22 |  | 24.95 | 22.43 | 20.18 | 22.52 | 2.38 |
| **180** | 27.23 | 32.78 | 27.73 | 29.25 | 3.07 |  | 31.86 | 32.15 | 25.53 | 29.85 | 3.74 |
| **240** | 33.70 | 27.63 | 26.90 | 29.41 | 3.74 |  | 28.62 | 37.75 | 28.35 | 31.57 | 5.35 |

***P*app calculations for the samples after 60min**

|  | **Apical to basal transport** | | | | **Basal to apical transport** | | | | | **Efflux ratio** | | | |
| --- | --- | --- | --- | --- | --- | --- | --- | --- | --- | --- | --- | --- | --- |
| **EFV** | Conc. (fmoles) | | *P*appAB (10^6^ cm/s) | | Conc. (fmoles) | | | *P*appBA (10^6^ cm/s) | | **ER** | **Mean** | **STD DEV** | ***p***  **value** |
| Sample # | Apical | Basal | *P*app | Mean | Basal | | Apical | *P*app | Mean |  |  |  |  |
| 1 | 181.93 | 8.30 | 5.42 | 4.51 | 103.18 | | 16.19 | 18.66 | 13.25 | 3.44 | 2.89 | 0.50 | 0.1822 |
| 2 | 236.90 | 7.46 | 3.74 |  | 180.85 | | 15.75 | 10.36 |  | 2.77 |  |  |  |
| 3 | 227.68 | 8.35 | 4.37 |  | 234.97 | | 21.16 | 10.71 |  | 2.45 |  |  |  |
| **EFV + IVM** | Conc. (fmoles) | | *P*appAB (10^6^ cm/s) | | Conc. (fmoles) | | | *P*appBA (10^6^ cm/s) | | **ER** | **Mean** | **STD DEV** |  |
| Sample # | Apical | Basal | *P*app | Mean | Basal | Apical | | *P*app | Mean |  |  |  |  |
| 1 | 178.22 | 14.84 | 9.91 | 6.73 | 167.54 | | 12.20 | 8.66 | 9.51 | 0.87 | 1.58 | 0.62 |  |
| 2 | 223.81 | 9.61 | 5.11 |  | 205.27 | | 15.87 | 9.20 |  | 1.80 |  |  |  |
| 3 | 210.16 | 9.16 | 5.18 |  | 180.34 | | 16.19 | 10.68 |  | 2.06 |  |  |  |
