# Supplementary material for: Drug-transporter mediated interactions between anthelminthic and antiretroviral drugs across the Caco-2 cell monolayers
Source: BMC Pharmacol Toxicol. 2017 May 4;18:20. doi: 10.1186/s40360-017-0129-6 (PMC5415745; doi:10.1186/s40360-017-0129-6)
Supplement: Supplementary file 9 — a Impact of LPV on the transport of IVM along the CCM. b Impact of IVM on the transport of LPV along the CCM. (ZIP 29 kb) [file 40360_2017_129_MOESM9_ESM.zip › Additional file 5b Impact of IVM on LPV along the CCMR3.docx]

**Impact of IVM on the transport of LPV along the CCM**

Apparent permeability coefficient (*P*app) expressed as mean ± S.D of three individual experiments (n=3)

**Cumulative transepithelial transport of [^3^H] LPV across the CCM alone, and in the presence of IVM**

| **LPV** | **Apical to basal transport (pmoles)** | | | | |  | **Basal to apical transport (pmoles)** | | | | |
| --- | --- | --- | --- | --- | --- | --- | --- | --- | --- | --- | --- |
| **Time(min)** | **1** | **2** | **3** | **Mean** | **STDEV** |  | **1** | **2** | **3** | **Mean** | **STDDEV** |
| **60** | 0.92 | 0.73 | 0.68 | 0.78 | 0.13 |  | 4.42 | 4.44 | 5.33 | 4.73 | 0.52 |
| **120** | 1.24 | 1.79 | 1.27 | 1.43 | 0.31 |  | 7.38 | 6.91 | 8.43 | 7.57 | 0.78 |
| **180** | 1.93 | 2.34 | 1.52 | 1.93 | 0.41 |  | 9.01 | 8.46 | 10.62 | 9.36 | 1.12 |
| **240** | 2.15 | 2.71 | 1.78 | 2.21 | 0.47 |  | 11.97 | 11.41 | 12.04 | 11.81 | 0.34 |
|  |  |  |  |  |  |  |  |  |  |  |  |
| **LPV + IVM** | **Apical to basal transport (pmoles)** | | | | |  | **Basal to apical transport (pmoles)** | | | | |
| **Time(min)** | **1** | **2** | **3** | **Mean** | **STDEV** |  | **1** | **2** | **3** | **Mean** | **STDEV** |
| **60** | 3.59 | 3.56 | 3.65 | 3.60 | 0.04 |  | 3.40 | 3.36 | 3.57 | 3.44 | 0.11 |
| **120** | 7.24 | 5.00 | 4.90 | 5.72 | 1.32 |  | 6.67 | 6.62 | 7.11 | 6.80 | 0.27 |
| **180** | 8.61 | 8.46 | 8.50 | 8.52 | 0.08 |  | 10.13 | 9.95 | 10.02 | 10.03 | 0.09 |
| **240** | 9.40 | 10.10 | 9.55 | 9.68 | 0.37 |  | 12.19 | 11.77 | 13.35 | 12.44 | 0.82 |

***P*app calculations for the samples after 60min**

|  | **Apical to basal transport** | | | | **Basal to apical transport** | | | | **Efflux ratio** | | | |
| --- | --- | --- | --- | --- | --- | --- | --- | --- | --- | --- | --- | --- |
| **LPV** | Conc. (pmoles) | | *P*appAB (10^9^ cm/s) | | Conc. (pmoles) | | *P*appBA (10^9^ cm/s) | | **ER** | **Mean ER** | **STD DEV** | ***p***  **value** |
| Sample # | Apical | Basal | *P*app | Mean | Basal | Apical | *P*app | Mean |  |  |  |  |
| 1 | 18.43 | 0.92 | 5.95 | 4.82 | 14.48 | 4.42 | 36.31 | 32.09 | 6.11 | 6.72 | 0.54 | 0.0038 |
| 2 | 19.89 | 0.73 | 4.38 |  | 17.27 | 4.44 | 30.59 |  | 6.99 |  |  |  |
| 3 | 19.61 | 0.68 | 4.15 |  | 21.60 | 5.33 | 29.38 |  | 7.08 |  |  |  |
| **LPV + IVM** | Conc. (pmoles) | | *P*appAB (10^6^ cm/s) | | Conc. (pmoles) | | *P*appBA (10^6^ cm/s) | | **ER** | **Mean ER** | **STD DEV** |  |
| Sample # | Apical | Basal | *P*app | Mean | Basal | Apical | *P*app | Mean |  |  |  |  |
| 1 | 29.66 | 3.59 | 14.38 | 15.95 | 31.40 | 3.40 | 12.87 | 12.29 | 0.89 | 0.78 | 0.10 |  |
| 2 | 27.79 | 3.56 | 15.25 |  | 35.85 | 3.36 | 11.14 |  | 0.73 |  |  |  |
| 3 | 23.83 | 3.65 | 18.21 |  | 33.01 | 3.57 | 12.87 |  | 0.71 |  |  |  |
