# Supplementary material for: Drug-transporter mediated interactions between anthelminthic and antiretroviral drugs across the Caco-2 cell monolayers
Source: BMC Pharmacol Toxicol. 2017 May 4;18:20. doi: 10.1186/s40360-017-0129-6 (PMC5415745; doi:10.1186/s40360-017-0129-6)
Supplement: Supplementary file 10 — a Impact of EFV on the transport of IVM along the CCM. b Impact of IVM on the transport of EFV along the CCM. (ZIP 28 kb) [file 40360_2017_129_MOESM10_ESM.zip › Additional file 6a Impact of EFV on IVM along the CCMR3.docx]

**Impact of EFV on the transport of IVM along the CCM**

Apparent permeability coefficient (*P*app) expressed as mean ± S.D of three individual experiments (n=3)

**Cumulative transepithelial transport of [^3^H] IVM across the CCM alone, and in the presence of EFV**

| **IVM** | **Apical to basal transport (pmoles)** | | | | |  | **Basal to apical transport (pmoles)** | | | | |
| --- | --- | --- | --- | --- | --- | --- | --- | --- | --- | --- | --- |
| **Time(min)** | **1** | **2** | **3** | **Mean** | **STDEV** |  | **1** | **2** | **3** | **Mean** | **STDDEV** |
| **60** | 0.57 | 0.44 | 1.00 | 0.67 | 0.30 |  | 0.65 | 0.55 | 0.96 | 0.72 | 0.22 |
| **120** | 0.50 | 0.61 | 1.00 | 0.71 | 0.26 |  | 1.34 | 1.05 | 1.67 | 1.36 | 0.31 |
| **180** | 0.66 | 0.77 | 1.54 | 0.99 | 0.48 |  | 1.49 | 1.45 | 1.96 | 1.63 | 0.28 |
| **240** | 0.96 | 0.81 | 1.30 | 1.03 | 0.25 |  | 2.52 | 1.59 | 2.27 | 2.13 | 0.48 |
|  |  |  |  |  |  |  |  |  |  |  |  |
| **IVM + EFV** | **Apical to basal transport (pmoles)** | | | | |  | **Basal to apical transport (pmoles)** | | | | |
| **Time(min)** | **1** | **2** | **3** | **Mean** | **STDEV** |  | **1** | **2** | **3** | **Mean** | **STDEV** |
| **60** | 0.54 | 0.41 | 0.33 | 0.43 | 0.11 |  | 0.46 | 0.72 | 1.29 | 0.82 | 0.43 |
| **120** | 0.83 | 0.68 | 0.48 | 0.66 | 0.18 |  | 1.09 | 0.99 | 0.62 | 0.90 | 0.25 |
| **180** | 0.96 | 0.75 | 0.86 | 0.85 | 0.11 |  | 1.36 | 1.32 | 1.12 | 1.26 | 0.13 |
| **240** | 1.29 | 0.86 | 1.40 | 1.18 | 0.28 |  | 1.52 | 1.52 | 1.41 | 1.49 | 0.06 |

***P*app calculations for the samples after 60min**

|  | **Apical to basal transport** | | | | **Basal to apical transport** | | | | **Efflux ratio** | | | |
| --- | --- | --- | --- | --- | --- | --- | --- | --- | --- | --- | --- | --- |
| **IVM** | Conc. (fmoles) | | *P*appAB (10^9^ cm/s) | | Conc. (fmoles) | | *P*appBA (10^9^ cm/s) | | **ER** | **Mean** | **STD DEV** | ***p***  **value** |
| Sample # | Apical | Basal | *P*app | Mean | Basal | Apical | *P*app | Mean |  |  |  |  |
| 1 | 8.33 | 0.57 | 7.59 | 7.60 | 6.96 | 0.65 | 10.18 | 8.94 | 1.34 | 1.19 | 0.15 | 0.4280 |
| 2 | 10.06 | 0.44 | 4.95 |  | 10.47 | 0.55 | 5.89 |  | 1.19 |  |  |  |
| 3 | 10.63 | 1.00 | 10.26 |  | 9.68 | 0.96 | 10.76 |  | 1.05 |  |  |  |
| **IVM + EFV** | Conc. (fmoles) | | *P*appAB (10^6^ cm/s) | | Conc. (fmoles) | | *P*appBA (10^6^ cm/s) | | **ER** | **Mean** | **STD DEV** |  |
| Sample # | Apical | Basal | *P*app | Mean | Basal | Apical | *P*app | Mean |  |  |  |  |
| 1 | 9.84 | 0.54 | 6.23 | 4.73 | 9.51 | 0.46 | 5.44 | 7.27 | 0.87 | 1.67 | 0.69 |  |
| 2 | 11.62 | 0.41 | 4.09 |  | 9.89 | 0.72 | 8.09 |  | 1.98 |  |  |  |
| 3 | 9.73 | 0.33 | 3.86 |  | 17.20 | 1.29 | 8.29 |  | 2.15 |  |  |  |
