# Supplementary material for: Drug-transporter mediated interactions between anthelminthic and antiretroviral drugs across the Caco-2 cell monolayers
Source: BMC Pharmacol Toxicol. 2017 May 4;18:20. doi: 10.1186/s40360-017-0129-6 (PMC5415745; doi:10.1186/s40360-017-0129-6)
Supplement: Supplementary file 10 — a Impact of EFV on the transport of IVM along the CCM. b Impact of IVM on the transport of EFV along the CCM. (ZIP 28 kb) [file 40360_2017_129_MOESM10_ESM.zip › Additional file 6b Impact of IVM on EFV along the CCMR3.docx]

**Impact of IVM on the transport of EFV along the CCM**

Apparent permeability coefficient (*P*app) expressed as mean ± S.D of three individual experiments (n=3)

**Cumulative transepithelial transport of [^14^C] EFV across the CCM alone, and in the presence of IVM**

| **EFV** | **Apical to basal transport (pmoles)** | | | | |  | **Basal to apical transport (pmoles)** | | | | |
| --- | --- | --- | --- | --- | --- | --- | --- | --- | --- | --- | --- |
| **Time(min)** | **1** | **2** | **3** | **Mean** | **STDEV** |  | **1** | **2** | **3** | **Mean** | **STDEV** |
| **60** | 0.31 | 0.32 | 0.31 | 0.32 | 0.00 |  | 0.30 | 0.25 | 0.30 | 0.28 | 0.03 |
| **120** | 0.61 | 0.54 | 0.55 | 0.56 | 0.04 |  | 0.53 | 0.50 | 0.57 | 0.53 | 0.04 |
| **180** | 0.67 | 0.70 | 0.71 | 0.69 | 0.02 |  | 0.69 | 0.65 | 0.70 | 0.68 | 0.03 |
| **240** | 0.78 | 0.86 | 0.82 | 0.82 | 0.04 |  | 0.86 | 0.78 | 0.84 | 0.83 | 0.04 |
|  |  |  |  |  |  |  |  |  |  |  |  |
| **EFV + IVM** | **Apical to basal transport (pmoles)** | | | | |  | **Basal to apical transport (pmoles)** | | | | |
| **Time(min)** | **1** | **2** | **3** | **Mean** | **STDEV** |  | **1** | **2** | **3** | **Mean** | **STDEV** |
| **60** | 0.21 | 0.20 | 0.22 | 0.21 | 0.01 |  | 0.25 | 0.27 | 0.28 | 0.27 | 0.01 |
| **120** | 0.52 | 0.63 | 0.59 | 0.58 | 0.06 |  | 0.52 | 0.52 | 0.59 | 0.54 | 0.04 |
| **180** | 0.72 | 0.60 | 0.62 | 0.64 | 0.07 |  | 0.70 | 0.71 | 0.77 | 0.72 | 0.04 |
| **240** | 0.87 | 0.78 | 0.76 | 0.80 | 0.06 |  | 0.85 | 0.81 | 0.91 | 0.85 | 0.05 |

***P*app calculations for the samples after 60min**

|  | **Apical to basal transport** | | | | **Basal to apical transport** | | | | **Efflux ratio** | | |  |
| --- | --- | --- | --- | --- | --- | --- | --- | --- | --- | --- | --- | --- |
| **EFV** | Conc. (pmoles) | | *P*appAB (10^6^ cm/s) | | Conc. (pmoles) | | *P*appBA (10^6^ cm/s) | | **ER** | **Mean** | **STD DEV** | ***p* value** |
| Sample # | Apical | Basal | *P*app | Mean | Basal | Apical | *P*app | Mean |  |  |  |  |
| 1 | 1.87 | 0.31 | 20.00 | 16.48 | 2.71 | 0.30 | 13.37 | 10.78 | 0.67 | 0.66 | 0.07 | 0.0310 |
| 2 | 1.97 | 0.32 | 15.91 |  | 2.68 | 0.25 | 9.29 |  | 0.58 |  |  |  |
| 3 | 1.93 | 0.31 | 13.52 |  | 2.55 | 0.30 | 9.67 |  | 0.72 |  |  |  |
| **EFV + IVM** | Conc. (pmoles) | | *P*appAB (10^6^ cm/s) | | Conc. (pmoles) | | *P*appBA (10^6^ cm/s) | | **ER** | **Mean** | **STD DEV** |  |
| Sample # | Apical | Basal | *P*app | Mean | Basal | Apical | *P*app | Mean |  |  |  |  |
| 1 | 2.31 | 0.21 | 10.73 | 8.56 | 3.20 | 0.25 | 9.29 | 7.69 | 0.87 | 0.90 | 0.03 |  |
| 2 | 2.44 | 0.20 | 7.98 |  | 3.60 | 0.27 | 7.33 |  | 0.92 |  |  |  |
| 3 | 2.58 | 0.22 | 6.96 |  | 3.57 | 0.28 | 6.45 |  | 0.93 |  |  |  |
